# The Prevalence of Symptomatic Knee Osteoarthritis in Relation to Age, Sex, Area, Region, and Body Mass Index in China: A Systematic Review and Meta-Analysis

**DOI:** 10.3389/fmed.2020.00304

**Published:** 2020-07-16

**Authors:** Danhui Li, Shengjie Li, Qiang Chen, Xuesheng Xie

**Affiliations:** ^1^Department of Pathology, Renji Hospital, School of Medicine, Shanghai Jiao Tong University, Shanghai, China; ^2^Department of Clinical Laboratory, Eye & ENT Hospital, Shanghai Medical College, Fudan University, Shanghai, China; ^3^School of Public Health, Shandong First Medical University, Tai'an, China; ^4^Department of Orthopaedics, Jinan City People's Hospital, Shandong, China

**Keywords:** osteoarthritis, prevalence, age, sex, area, region

## Abstract

**Purpose:** This study aimed to investigate the overall prevalence of symptomatic knee osteoarthritis (OA) in China by conducting a meta-analysis.

**Methods:** Six databases were searched for articles published from the date of inception to October 1, 2017, based on the Population, Intervention, Comparator, Outcomes (PICO) framework. The review was in line with preferred reporting items for systematic reviews and meta-analyses (PRISMA) guidelines. The χ^2^-based Q statistic and *I*^2^ metrics were used for exploring the sources of heterogeneity. Random models were utilized to obtain prevalence estimates due to the heterogeneity that was observed. Comprehensive Meta-Analysis version 2.0 was used for assessing publication bias by inspecting funnel plots and Egger's tests.

**Results:** Twenty-one eligible studies (74,908 participants in total) were identified. The overall pooled prevalence of symptomatic knee OA in China was 14.6%. The prevalence of symptomatic knee OA presented a rapid growth trend between the periods of 1990–2008 and 2008–2013 (9.1 vs. 20.1%, *p* = 0.005). However, after 2013, the prevalence dropped to 14.9% (*p* = 0.01). The prevalence rates of symptomatic knee OA increased with age and presented an almost linear growth after 40 years of age. Compared with males (10.9%), females (19.1%) exhibited a higher prevalence of symptomatic knee OA (*p* = 0.015). The symptomatic knee OA prevalence was significantly higher in rural than it was in urban areas (16.9 vs. 11.1%, *p* = 0.037).

**Conclusion:** For symptomatic knee OA intervention, more attention should be paid to females, people in rural areas, and people aged over 40 years.

## Introduction

Osteoarthritis (OA) is a chronic disease occurring in different parts of the body; it is characterized by cartilage destruction, subchondral bone sclerosis, and arthritic bone hyperplasia (1). Knee OA, the main type of OA, is ranked as the 11th highest contributor to global disability and 38th highest in disability-adjusted life years (2). A number of factors may contribute to the development of knee OA, including prior joint injury, obesity, sex, and anatomical factors related to joint shape and alignment (3). There is no cure for knee OA, and treatment is directed at symptom relief. OA's current effect on society is tremendous. The estimated lifetime risk is 13.83% among New Zealand adults (4), and knee OA is responsible for heavy losses of lifetime quality-adjusted life years (5).

Symptomatic OA, radiographic OA, and self-reported OA are the most commonly used case definitions (6). OA cases are defined as symptomatic when both radiographic and joint symptoms related to the pathology are present (7). In most research, the preferred definition for epidemiological studies is symptomatic OA (6). To improve the comparability with other countries globally, we adopted universal standards and systematically analyzed the prevalence of symptomatic knee OA in China. However, epidemiological research on symptomatic knee OA is challenged by some specific problems, including different possibly affected joint sites with different pathological patterns and the need for radiographic examination for clinical confirmation (8). Other systematic reviews have been conducted on the association of gene polymorphism, with its susceptibility to OA (9, 10), and the comparative effectiveness of certain medicines (11). However, a systematic review of the prevalence of symptomatic knee OA in the Chinese population is lacking. China comprises 34 province-level administrative regions with a population of 1.4 billion people. A country-wide survey of the prevalence of symptomatic knee OA in China is necessary and the ideal study design to assess the prevalence of OA should be a nationwide population study. Of course this is not really feasible, so a systematic review and meta-analysis represents a good surrogate. In this review, we investigate the overall prevalence of symptomatic knee OA in China and assess the prevalence of knee OA in different subgroups for age, sex, study year, country area, country region, and body mass index (BMI) by conducting a meta-analysis.

## Methods

The review was in line with preferred reporting items for systematic reviews and meta-analyses (PRISMA) guidelines.

### Search Strategy

PubMed, EMBASE, the Web of Science, the China National Knowledge Infrastructure, the VIP Database for Chinese Technical Periodicals, and the Wan Fang Database for Chinese Periodicals were electronically searched by two authors (Danhui Li and Shengjie Li) to identify studies published from the date of inception to October 1, 2017. The search terms included “osteoarthritis,” “prevalence or incidence or epidemiology,” AND “China or Chinese.” Varying combinations of the search terms were used for identifying relevant literature, and the search strategies were customized to suit each database (Supplementary Table 1). Reference lists of eligible articles were also retrieved for identifying potential studies.

### Inclusion Criteria

The clinical PICO question was as follows:

1. Studies involving patients on symptomatic knee OA in China;
2. Studies recording data on the prevalence by age, sex, region, city, and year;
3. The sample size of studies was >500;
4. The type of study: random or cluster sampling;
5. Studies conducted in special groups (pregnant women, diabetics, cardiovascular disease patients) were excluded.

### Data Extraction

Note Express was implemented to remove duplicates. The titles and abstracts of all studies were screened independently by two reviewers (Danhui Li and Qiang Chen) against the inclusion and exclusion criteria. Articles that did not meet the inclusion criteria were excluded. Full-text articles were evaluated for eligibility. The remaining studies were included in the meta-analysis. Any disagreement was settled by a third reviewer. One investigator extracted data, which included (1) first author; (2) study year; (3) province; (4) average age; (5) region (urban or rural); (6) response rate (the ratio of sample size to the sample size of the planned survey); (7) overall sample size; (8) the method used to sample subjects; (9) diagnostic criteria of symptomatic knee OA; (10) the prevalence rates in different subgroups of BMI; (11) the prevalence rates of the overall sample in different age ranges; and (12) the prevalence rates of males and females in different age ranges.

### Quality Assessment

The tool adopted to assess quality was adapted from the references of previous prevalence studies (12, 13). Six quality assessment criteria were employed for assessment of the included studies, where the following questions were asked:

1. Was the study design clearly described?
2. Did the study define the information source of the survey?
3. Did the study report the period of patient inclusion?
4. Were the age and sex of eligible participants clearly described?
5. Did the study summarize the patient response rates and completeness of the data collection?
6. Did the study clearly report the diagnostic criteria of symptomatic knee OA?

In addressing these questions, a response of “clear or adequate” was scored as 1 point, whereas “no or not available” was scored as 0 points. A maximum score of 6 was possible for each study. The greater the quality score, the more adequate the quality of the study was considered to be.

### Statistical Analysis

Comprehensive Meta-Analysis version 2.0 (Biostat, Englewood Cliffs, NJ, USA; http://www.meta-analysis.com) was applied for estimating the prevalence rates of symptomatic knee OA, with 95% confidence intervals (CIs) overall and by subgroup. Heterogeneity was tested with the χ^2^-based Q statistic and *I*^2^ statistic. The smaller the *I*^2^ statistic, the smaller the heterogeneity. A value of *I*^2^ > 50% indicated a high level of heterogeneity. A random-effects model was used to calculate whether heterogeneity was observed (*p* < 0.05); otherwise, a fixed-effects model was applied if there was no heterogeneity (14). We performed subgroup analysis by year of data collection (1990 ≤ year ≤ 2008, 2008 < year ≤ 2013, and year > 2013), region (urban and rural), area (southern and northern China), sex (female and male), age group (15–39, 40–49, 50–59, 60–69, and over 70 years old), and separately for males, females, and overall. The statistically significant differences between the subgroups were reviewed using the Kruskal–Wallis and Mann–Whitney *U*-tests. The *Z*-value and *p*-value were obtained, and a value of *p* < 0.05 was considered statistically significant. To examine the authenticity of the data, Egger's tests and funnel plots were generated using Comprehensive Meta-Analysis version 2.0. No publication bias exists if the studies are arranged symmetrically around the central line with a *p*-value of >0.05 (15).

## Results

### Characteristics of the Studies

Using the initial search strategy, we identified 1,268 records. After 723 duplicates were removed, the titles and abstracts of 545 studies were screened. For diverse reasons, 475 records were excluded, and the remaining 70 full texts were assessed using the inclusion criteria. Finally, we included 21 studies incorporating 74,908 Chinese individuals in the meta-analysis. A flow chart of the article search process is shown in Supplementary Figure 1. The detailed characteristics of 21 studies (16–36) are shown in Table 1. The overall quality of the included studies was considered acceptable (Supplementary Table 2). The overall mean score for the quality of the studies included in the analysis regarding the prevalence of symptomatic knee OA was 4.86. The period of patient inclusion was the poorest reported on. Information on how OA was diagnosed for each study is included in Supplementary Table 3. A random-effects model was chosen for the analysis due to the significant heterogeneity in this meta-analysis (Supplementary Table 4).

**Table 1:** Characteristics of the populations examined in studies reporting the prevalence of symptomatic knee osteoarthritis in mainland China.

**Name**			**Junfeng Zhang**	**Keqiang Huang**	**Xu Tang**			**Yuan Liu**	**Yuewen Wang**	**Liying Jiang**		
Year			2013	2013	2016			2016	2013	2012		
Province			Shanxi	Guang dong	N/A			Shanghai	Inner Mongolia	Heilongjiang		
Average age			43.9 ± 16.6	≥65	59.8			40–74	58	62.6 ± 8.69		
Region			Rural	Hospital	Total	Rural	Urban	Rural	Urban	Total	Rural	Urban
Response rate (%)			N/A	N/A	N/A			75.6	93.85	99.67		
Sample size			7,126	1,534	17,128	1,0206	6,922	3,428	1,054	1,196	595	601
BMI	≤ 24		15	N/A	N/A			N/A	N/A	12.7	12.2	13.7
	24–28		20.7							20.0	17.1	21.7
	≥28		27.6							28.7	22.7	30.0
Prevalence (%)	Male	15–39	6.20									
		40–49	26.52		(<50)3.3			9.09		5.54		
		50–59	38.13		5.7			19.32		12.57		
		60–69	41.8		7.5			20.04		25.58		
		≥70	48.14		5.9			24.71		25.81		
		Total	23	9.95	5.7	8.3	2.9	17.4		11.91	12.31	11.48
	Female	15–39	6.85									
		40–49	28.14		(<50)6.8			9.59		10.75		
		50–59	40.95		9.1			17.25		23.9		
		60–69	50.7		14.7			21.45		37.78		
		≥70	57.41		11.5			29.25		41.18		
		Total	25.7	37.77	10.3	13.5	7.2	15.79		19.87	14.68	26.01
	Total	15–39	6.52									
		40–49	16.7		(<50)5.2			9.4		8.3		
		50–59	39.71		7.4			18.26		18.56		
		60–69	45.83		11.1			20.64		31.82		
		≥70	52.84		(≥70)8.8			26.45		31.25		
		Total	24.3	17.4	8.1	10.9	5.2	16.57	12.43	16.05	13.61	12.49
**Name**			**Xiaozheng Kang**	**Hui Du**	**Yanfeng Zhang**	**Shaoqi Tian**			**Qingyun Xue**	**Yufei Li**	**Xiaowei Xiang**	
Year			2009	2005	2016	2015			2015	2015	2014	
Province			Inner Mongolia	Shanghai	Xinjiang	Shandong			6 cities	Hunan	Guangdong	
Average age			64 ± 7	≥40	38	62.2 ± 1.5			54.82 ± 9.96	N/A	N/A	
Region			Rural	Urban	Rural	Total	Rural	Urban	Total	Rural	Urban	
Response rate (%)			91	90.8	96.12	90.87			N/A	93.1	N/A	
Sample size			1,030	2,093	1,461	1,125	555	570	6,218	1,263	1,000	
BMI	≤ 24		N/A	N/A	N/A	9.57			N/A	71.2	N/A	
	24–28					16.12				18.4		
	≥28					24.78				11.4		
Prevalence (%)	Male	15–39										
		40–49			8.36	1.8						
		50–59	3		15.96	3					7.74	
		60–69	13			9.9					5.58	
		≥70	18			18.7					10.05	
		Total	7	3.7	12.83	8.5				16.6	7	
	Female	15–39										
		40–49			27.19	5.2						
		50–59	9		32.83	9.7					6.58	
		60–69	29			22.5					7.94	
		≥70	31			41.3					19.05	
		Total	14	9.8	34.1	20.3				23.4	12.4	
	Total	15–39										
		40–49		1.3	17.91	3.5						
		50–59	5.93		25.83	6.5					5.19	
		60–69	19.9			16.5					6.71	
		≥70	23.28	13.2		30.65					14.79	
		Total	10.63	7.2	24.23	14.7	15.3	14	15.6	20.3	9.7	
**Name**			**Zhenyong Xiang**	**Bin Gu**	**Yan Fan**	**Lei Wang**	**Minsheng Tang**	**Wei Wang**			**Changhai Zang**	**Qingyu Zeng**
Year			2013	2011	2010	2008	2007	2007			2006	2006
Province			Shanghai	Shanghai	Shanghai	Tianjin	Guangdong	Shanxi			Shanxi	Shanxi
Average age			N/A	62.3 ± 11.1	N/A	63.4	45.60 ± 12.23	54.4			46 ± 16	
Region			Rural	Rural	Urban	Urban	Urban	Total	Rural	Urban	Urban	Urban
Response rate (%)			78.6	78.6	N/A	N/A	N/A	N/A			97.75	N/A
Sample size			3343	1499	1002	2235	13110	923	426	497	3915	2188
BMI	≤ 24		28.4	N/A	N/A	15.02	N/A	N/A			N/A	N/A
	24–28		39.0			16.60						
	≥28		52.2			32.08						
Prevalence (%)	Male	15–39									1.20	
		40–49	6.5			2.5					5.02	
		50–59	16.2			3.8					10.93	
		60–69	26.9	5.31	25.33	8.5					14.14	
		≥70	39	14.02	43.15	18.81					21.79	
		Total	24	11.9	33.73	9.1	2.52		4.7	5.9	7.59	18.3
	Female	15–39									1.63	
		40–49	10.8			6.4					12.57	
		50–59	35			15.8					26.91	
		60–69	52.4	6.59	37.14	20					38.83	
		≥70	51.4	26.23	52.63	29.67					33.21	
		Total	41.6	21	45.2	19.4	6.04		13	13.8	16.19	8.7
	Total	15–39									1.42	
		40–49	8.5			4.3		4.6			9.12	
		50–59	26.7			9.3		8.4			18.15	
		60–69	39.4	6.01	31.94	13.9		13.7			30.31	
		≥70	45.7	21.17	48.9	24.38		24.3			27.68	
		Total	33.3	17	40.42	14	4.26	9.5	8.9	10.1	12.11	10.9

### Overall Prevalence Rates of Symptomatic Knee OA in Mainland China

**Figure 1:**
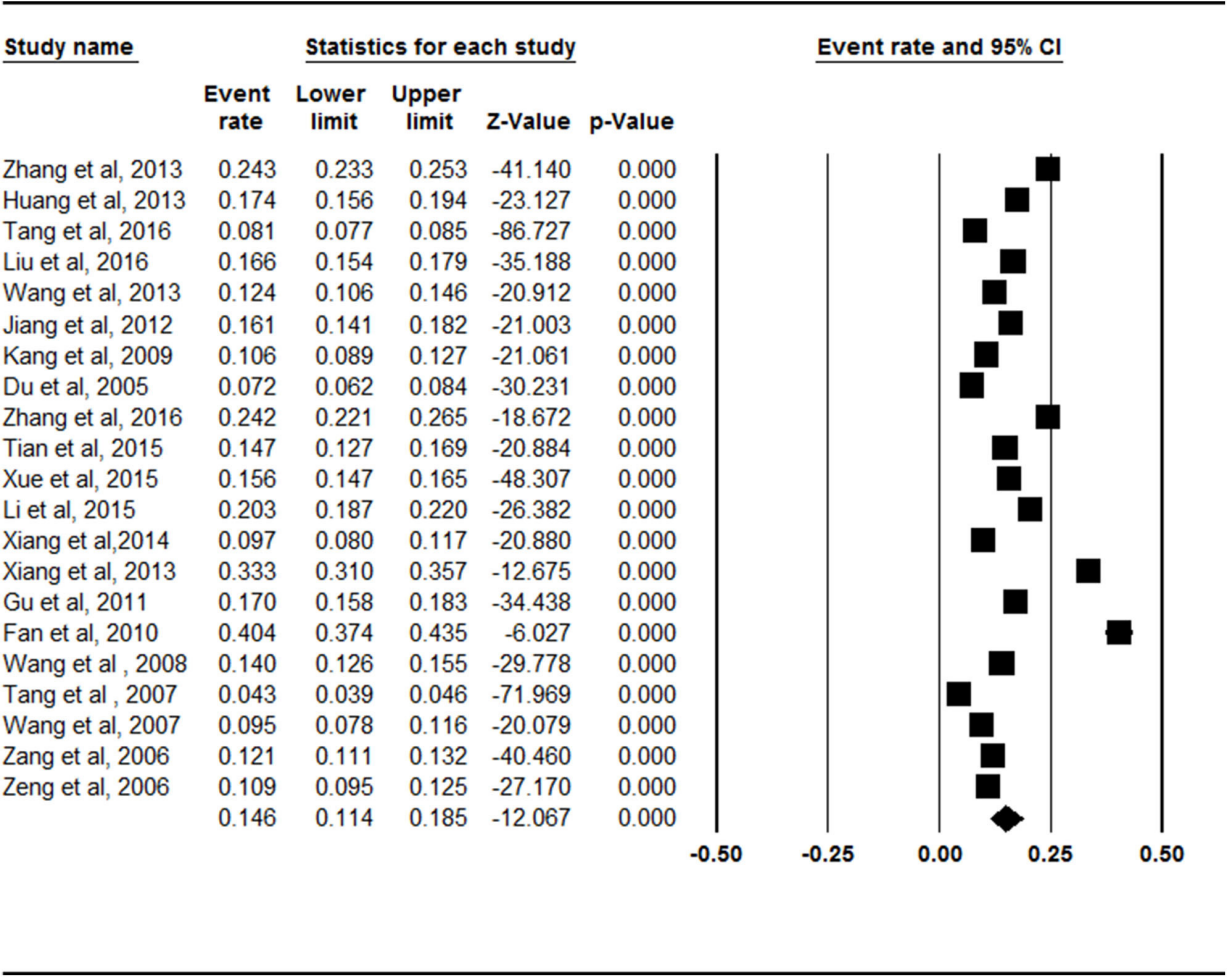
Forest plot of the overall prevalence rates of symptomatic knee osteoarthritis. CI, confidence interval. A total of 21 studies were included in this meta-analysis. The overall pooled prevalence of symptomatic knee osteoarthritis was calculated as 14.6% (95% CI = 0.114–0.185) through analyzing in a random-effects model.

The meta-analysis of the total prevalence estimates of the studies (*n* = 21, *N* = 74,908) showed that the overall pooled prevalence of symptomatic knee OA was 14.6% (95% CI = 11.4–18.5%, Figure 1).

### Prevalence Rates of Symptomatic Knee OA in Mainland China by Study Year

The prevalence of symptomatic knee OA presented a rapid growth trend between the period of 1990–2008 (9.1%, 95% CI = 5.8–13.9%) and 2008–2013 (20.1%, 95% CI = 15.1–26.1%), which was statistically significant (*Z* = −2.777, *p* = 0.005). However, after 2013, the prevalence dropped to 14.9% (95% CI = 10.6–20.4%), which was statistically significant (*Z* = −2.582, *p* = 0.01, Supplementary Figure 2).

### Prevalence Rates of Symptomatic Knee OA in Mainland China by Age

**Figure 2:**
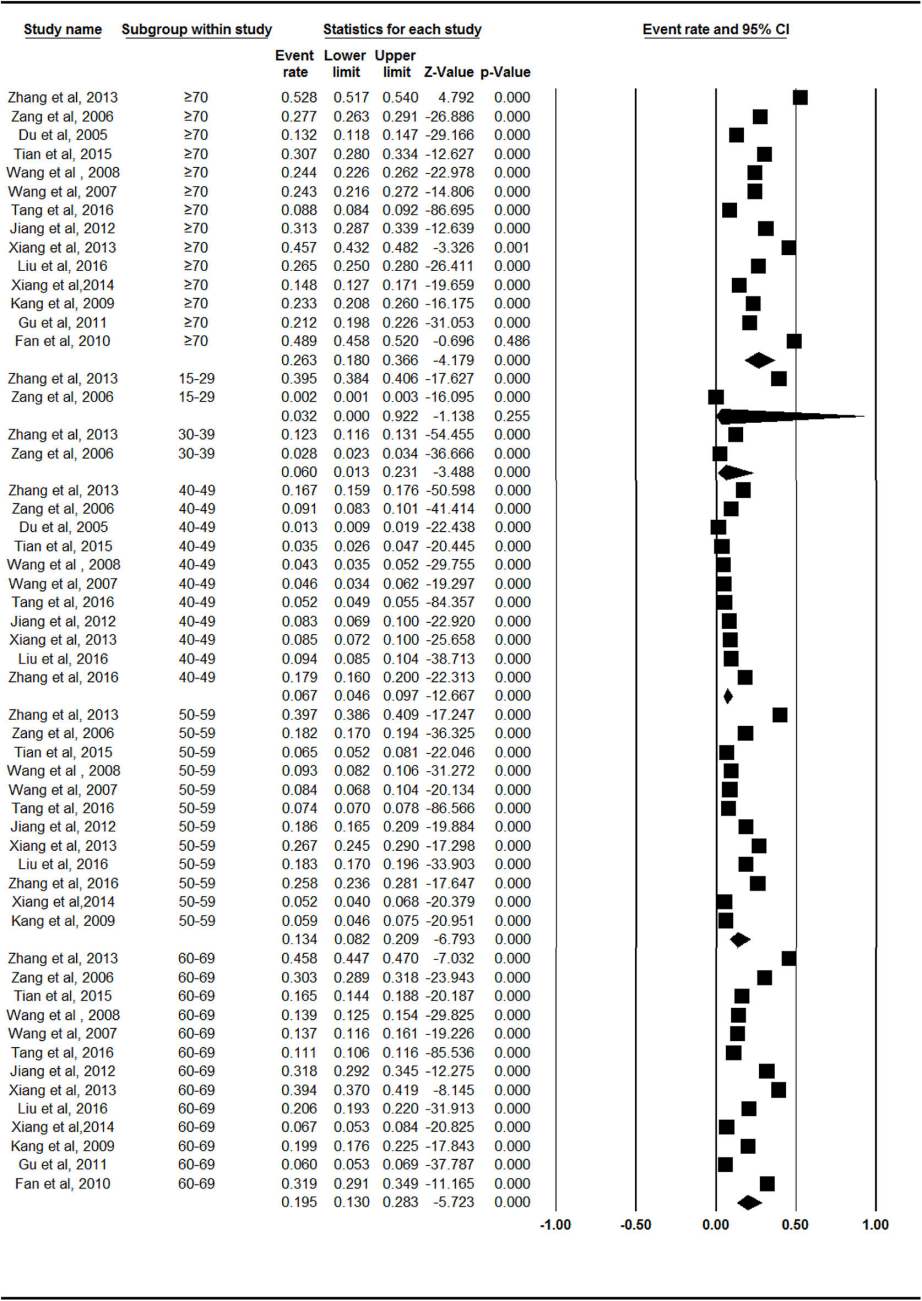
Forest plot of the prevalence rates of symptomatic knee osteoarthritis by age. CI, confidence interval. The prevalence of symptomatic knee osteoarthritis in different age group of 15–39 years old (*n* = 2), 40–49 years old (*n* = 11), 50–59 years old (*n* = 12), 60–69 years old (*n* = 13), over 70 years old (*n* = 14), respectively, were investigated. The prevalence rates of symptomatic knee osteoarthritis was evaluated as follows: 3.1% (95% CI = 0.7–13.0%) in 15–39 years old, 6.7% (95% CI = 4.6–9.7%) in 40–49 years old, 13.4% (95% CI = 8.2–20.9%) in 50–59 years old, 19.5% (95% CI = 13.0–28.3%) in 60–69 years old, 26.3% (95% CI = 18.0–36.6%) in age group over 70 years old.

The prevalence rates of symptomatic knee OA increased with age. The rate was lowest (3.1%, 95% CI = 0.7–13.0%) in the 15- to 39-year-old age group and highest (26.3%, 95% CI = 18.0–36.6%) in the group over 70 years of age (Figure 2, Supplementary Figure 3). There were statistically significant differences between Groups 2 and 3 (*Z* = −2.062, *p* = 0.039). There were no statistically significant differences between Groups 3 and 4 (*Z* = −1.366, *p* = 0.172) or Groups 4 and 5 (*Z* = −1.397, *p* = 0.163).

### Prevalence Rates of Symptomatic Knee OA in Mainland China by Sex

**Figure 3:**
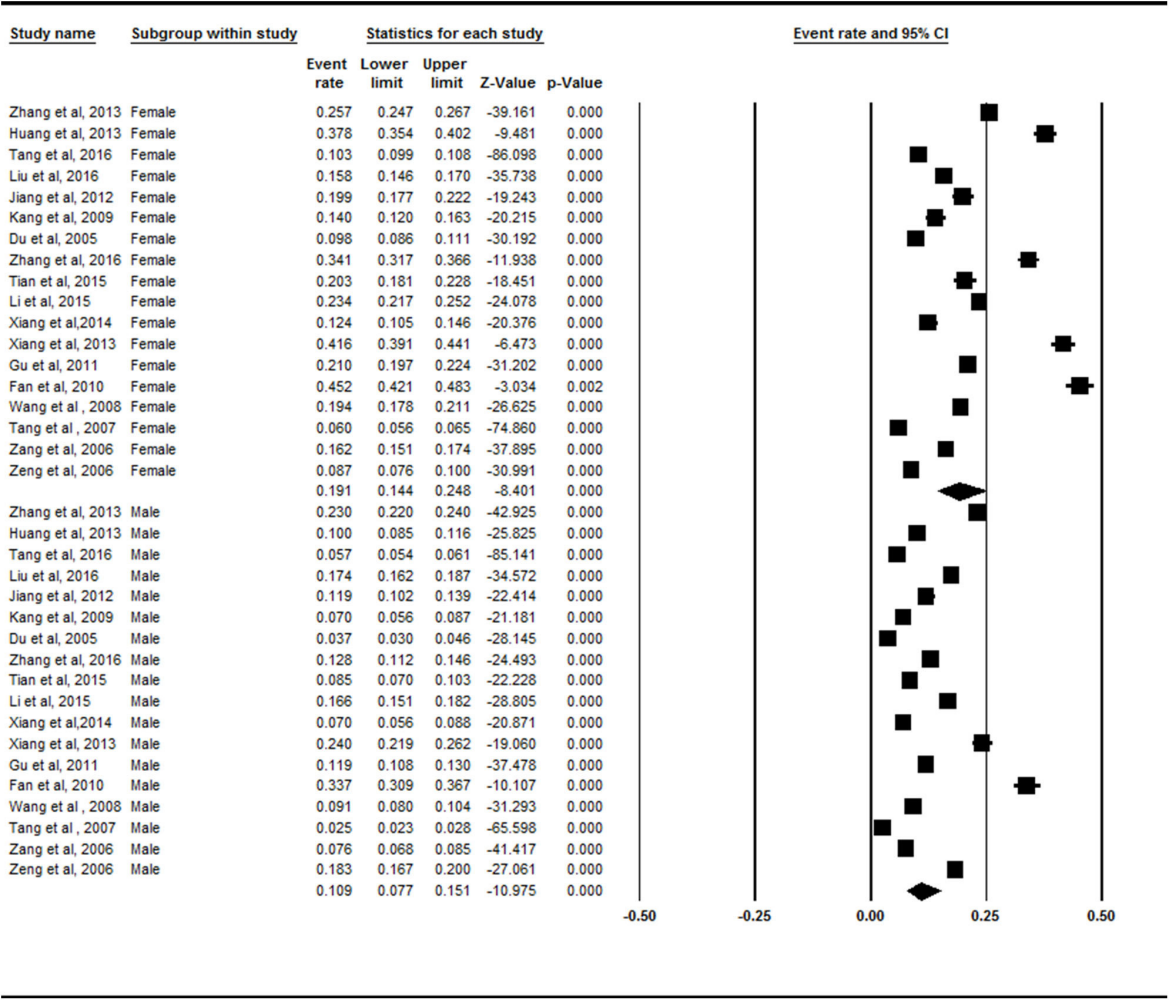
Forest plot of the prevalence rates of symptomatic knee osteoarthritis by sex. CI, confidence interval. A total of 18 studies investigated the different prevalence of symptomatic knee osteoarthritis in both sexes. Females (19.1%, 95% CI = 14.4–24.8) exhibited a higher prevalence of symptomatic knee osteoarthritis than males (10.9%, 95% CI = 7.7–15.1%) in the subgroup analysis.

Females (19.1%, 95% CI = 14.4–24.8) exhibited a higher prevalence of symptomatic knee OA than males did (10.9%, 95% CI = 7.7–15.1%) in the subgroup analysis (Figure 3). There was a statistically significant difference between the female and male subgroups (*Z* = −2.436, *p* = 0.015).

### Prevalence Rates of Symptomatic Knee OA in Mainland China by Age Divided Into Males and Females

We evaluated the prevalence values of all the age groups divided according to sex. The prevalence levels of symptomatic knee OA among age subgroups in males and females were both statistically significant (*Z* = 12.399, *p* < 0.001; *Z* = 48.261, *p* < 0.001). The prevalence rates of symptomatic knee OA increased with age in both sexes, and females showed more rapid increases compared with males, especially after 40 years old (Supplementary Figures 3–5).

### Prevalence Rates of Symptomatic Knee OA in Mainland China by Area

**Figure 4:**
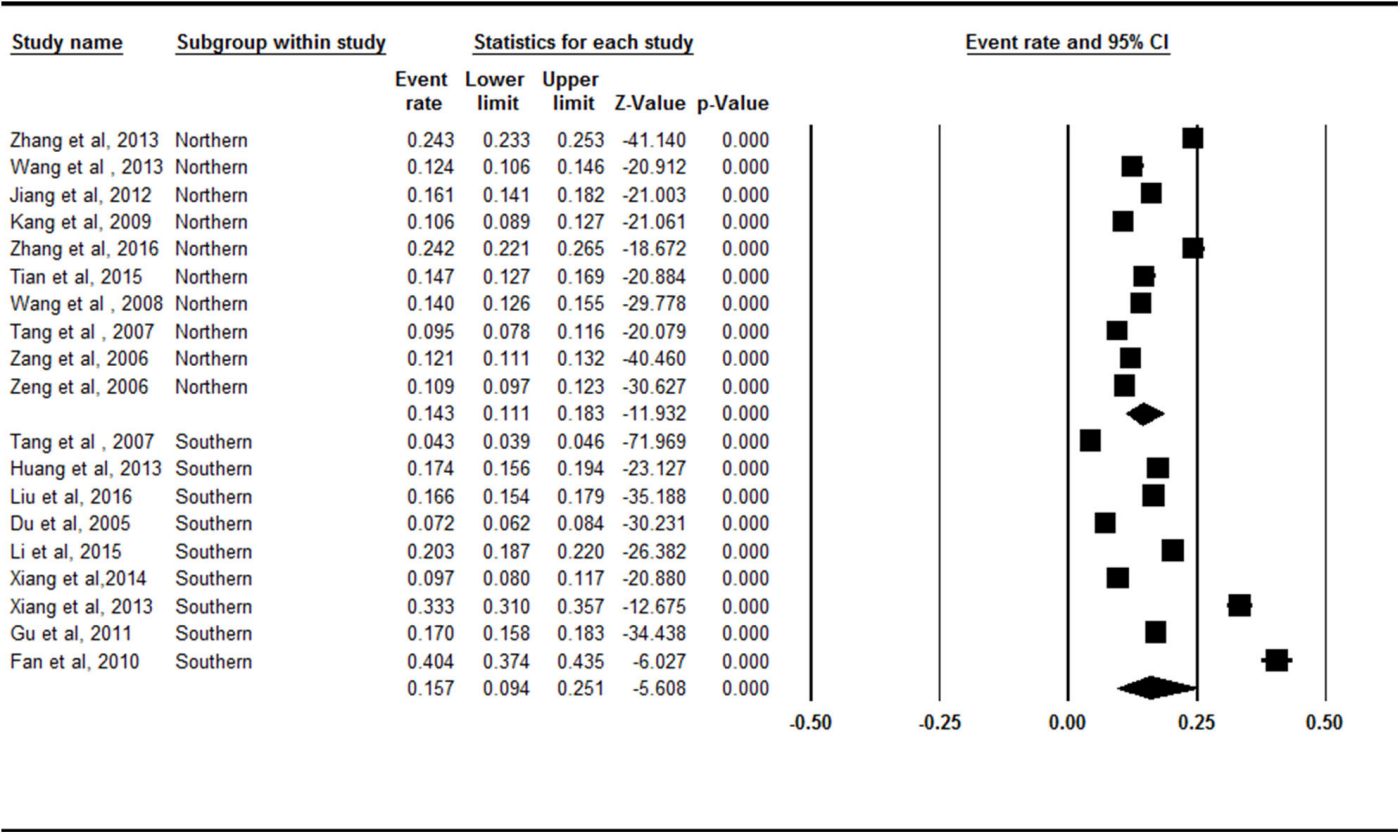
Forest plot of the prevalence rates of symptomatic knee osteoarthritis by area. CI, confidence interval. A total of 19 studies were included in this subgroup meta-analysis. Ten of these studies were conducted in Northern China and 9 studies were conducted in Southern China. The South (15.7%, 95% CI = 9.4–25.1%) had a higher prevalence of symptomatic knee osteoarthritis than the North (14.3%, 95% CI = 11.1–18.3%) in the subgroup analysis.

There was no statistically significant difference between the northern China (14.3%, 95% CI = 11.1–18.3%) and southern China (15.7%, 95% CI = 9.4–25.1%) subgroups (*Z* = −0.653, *p* = 0.514; Figure 4).

### Prevalence Rates of Symptomatic Knee OA in Mainland China by Region

**Figure 5:**
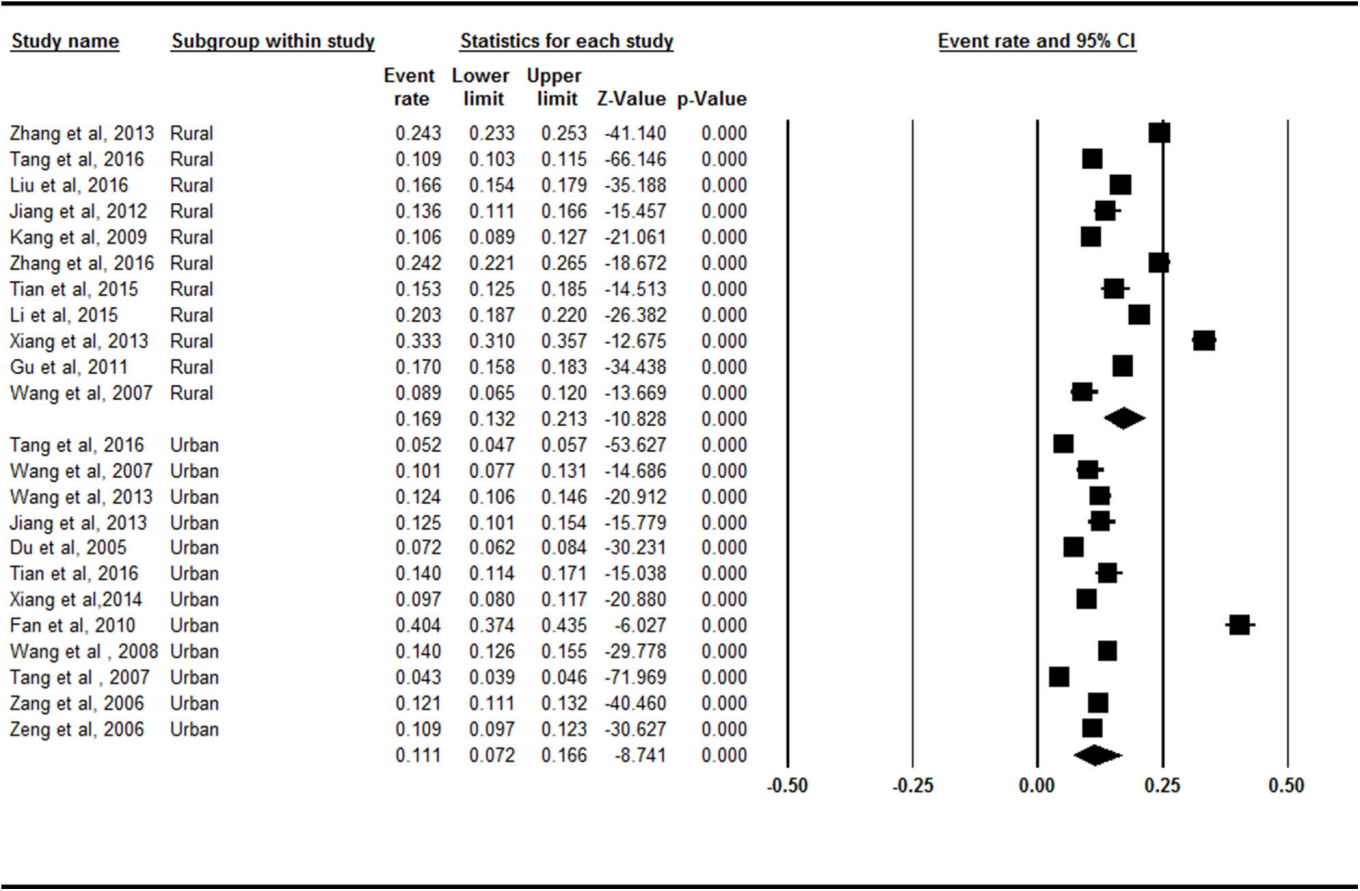
Forest plot of the prevalence rates of symptomatic knee osteoarthritis by region. CI, confidence interval. Eleven and 12 studies, respectively, investigated the prevalence of symptomatic knee osteoarthritis in rural and urban. The rural (16.9%, 95% CI = 13.2–21.3%) had a higher prevalence of symptomatic knee osteoarthritis than the urban (11.1%, 95% CI = 7.2–16.6) in the subgroup analysis.

The rural region (16.9%, 95% CI = 13.2–21.3%) had a higher prevalence of symptomatic knee OA than the urban region did (11.1%, 95% CI = 7.2–16.6) in the subgroup analysis (Figure 5). There was a statistically significant difference between the rural region and urban region subgroups (*Z* = −2.087, *p* = 0.037).

### Prevalence Rates of Symptomatic Knee OA in Mainland China by BMI

The prevalence rates of symptomatic knee OA were 28% (95% CI = 18.6–29.4%) in the obese, 21.1% (95% CI = 14.9–29.0%) in the overweight, and 15.3% (95% CI = 10.4–22%) in the normal weight groups (Supplementary Figure 6). There were no statistically significant differences between the BMI ≤ 24 and 24 < BMI <28 subgroups (*Z* = −1.830, *p* = 0.067), 24 < BMI <28 and BMI ≥ 28 subgroups (*Z* = −1.281, *p* = 0.2), or BMI ≤ 24 and BMI ≥ 28 subgroups (*Z* = −1.647, *p* = 0. 1).

### Analysis of Publication Bias

There were no obvious asymmetries in the funnel plots and the value of *p* > 0.05 for most groups, except the northern China subgroup (Supplementary Table 5, Supplementary Figures 7–23).

## Discussion

This was the first systematic review and meta-analysis focusing on the evaluation of the prevalence of symptomatic knee OA in China. In the meta-analysis, 21 studies (74,908 people) on symptomatic knee OA were included. The overall pooled prevalence of symptomatic knee OA in China was systematically evaluated, showing a moderate level of 14.6%. In previous research, the overall prevalence of symptomatic knee OA was found to be 15.4% in southern Sweden (37), while it was 10.5% in Canada (38). The different epidemiologies in different countries may be attributed to the difference in samples. The respondents for the southern Sweden study were a random sample of 10,000 residents of Malmo, Sweden, aged 56–84 years. A large adult population sample of individuals ≥18 years in four communities in the province of Alberta, Canada, was randomly sampled in the Canadian study. Furthermore, a major issue was that the definition of OA may have differed in the studies. Although the two studies illustrated the diagnostic procedure, the specific diagnostic criteria were not stated. The reliability of the radiological classification systems and exclusion criteria also differed in these studies. These unified samples and methods have yet to be established. They are the objective foundations of comparability of the prevalence between different countries.

The accelerated aging in China necessitates the establishment of a comprehensive social security system for the aging population. The prevalence rates of symptomatic knee OA increase with age, regardless of sex. The rates increase linearly with age after 40 years old, which is consistent with Kopec et al. (7). Aging and OA are closely related, and the condition is not reversible (6), which may contribute to the high prevalence of symptomatic knee OA in older people. However, it have been elucidated that aging contributed to OA by several potential mechanisms, as follows: age-related inflammation, oxidative stress, and dysfunction in energy metabolism (3). In both men and women, knee extensor muscle weakness led to an increased risk of developing knee OA (39). The knee extensors work as shock absorbers and stabilizers, and hence, they protect the joint surfaces during loading and movement (40). Excessive mechanical stress on articular cartilage due to muscle weakness has been suggested to induce a degenerative process (41, 42). In the elderly, muscles become atrophic and weaker in muscle mass and force. The muscles were more susceptible to damage in the elderly, while they regenerate and recover more slowly than occurred in youth (43). With longer life expectancy, the demographics predict an increase of aging-related OA of the knee for the next decade (44). This progressive condition may result in a substantial increase in the cost to society for health care.

The prevalence of symptomatic knee OA was higher in women of all age groups, which was consistent with Kopec and Yoshimura (7). Female sex hormones play a vital role in the etiology and pathophysiology of musculoskeletal degenerative diseases. After deleting estrogen receptors in female mice, studies have found that estrogen plays a protective role in the maintenance of joint homeostasis by preventing cartilage damage, osteophytosis, and changes in the subchondral bone of the joints (45). Most women reach menopause at 45–55 years of age, but it may occur between 40 and 60 years of age (46). Menopause is a period of transition from reproductive to non-reproductive life, with a corresponding reduction of estrogen (47). The drop in estrogen may contribute to the high prevalence of symptomatic knee OA in women. Males account for a larger proportion of drinking and smoking in China than females do (48). However, an inverse association was observed between cigarette smoking and knee OA in the linear trend test in the Chinese population (49).

There was not a statistically significant difference between northern China and southern China. The geography of China was divided into the south and north in relation to the Qinling Mountains and Huaihe River, which act as the climate line. The included articles did not cover all the cities in China, and the geographical distribution of the included studies is unbalanced. Five included studies were carried out in Shanghai. More extensive studies are needed to investigate the difference between northern China and southern China in terms of the OA prevalence.

The rural region had a higher prevalence of symptomatic knee OA than the urban region did. Occupation is an additional risk factor that has been linked to OA. Tang et al. (21) reported that occupational hazards (physical overloads), work history in one specialty for over 5 years, functional and static-dynamic loads on the bones and joints, and elevated temperature and humidity in the industrial premises were the main industrial risk factors for OA. Several studies showed that there is an increased risk of OA among construction workers (50), floor layers (51), and farmers and healthcare assistants (52). People in the rural regions of China make up the main group of those performing jobs that are harmful to the joints. Furthermore, people in rural regions tend not to be as well-informed as urban people about non-pharmacological core management of knee OA concerns, including patient information, education, lifestyle, exercise, weight loss, assistive technology, and adaptations (53). In addition, reports indicate that dietary antioxidants and dietary vitamin C intake are positively correlated with the prevalence of radiographic knee OA (54). The primary goals of the current OA therapy are not reversing the disease process, but centered on controlling pain, improving joint function, and improving health-related quality of life (55). However, unsound medical conditions and inadequate health awareness likely contribute to the high prevalence of symptomatic knee OA in rural communities.

The prevalence of symptomatic knee OA presented a rapid growth trend between the periods of 1990–2008 (9.1%) and 2008–2013 (20.1%); however, the prevalence dropped to 14.9% after 2013. In 1990–2008, the serious aging problem and economic crisis had a great effect on development in China. As reported in 2010, there were 111 million (8.2% of China's population) elderly Chinese individuals (aged 65+ years) (56). The prevalence rates of symptomatic knee OA increased with age. The accelerated aging resulted in an increased prevalence of symptomatic knee OA. However, by 2012, 262 million people had migrated to urban areas (57). The development of society has exacerbated momentous changes in the modes of production and lifestyle, and manual labor affects the prevalence of symptomatic knee OA (50–52).

The prevalence of symptomatic knee OA did not increase significantly as BMI increased. In the subgroup of BMI ≤ 24, most results were in the range of 9.57–28.4. An outlier (71.2%), the study conducted by Li et al. (17), was abnormal; thus, it was removed from the subgroup analysis. The possible reason for its outlier status may have been printing mistakes or errors in the experimentation.

Some limitations of this study should be considered. First, the tool adopted to assess quality was adapted from the references of previous prevalence studies. The fact of adopting a customized tool might be regarded as a limitation. Second, the included studies did not cover all the cities in China equally. Third, due to the heterogeneity among the papers, our sample changed from variable to variable. A large epidemiological investigation of symptomatic knee OA still needs to be carried out, and the association between OA and other diseases should be discussed in future investigations.

## Conclusion

The overall pooled prevalence of symptomatic knee OA was 14.6% (95% CI = 11.4–18.5%) in China. The prevalence rates of symptomatic knee OA presented an almost linear growth after 40 years old. Females exhibited a higher prevalence of symptomatic knee OA than males did, while the prevalence was significantly higher in rural than it was in urban areas. There was no statistically significant difference between northern China and southern China. The prevalence of symptomatic knee OA did not rise significantly as the BMI increased. More attention should be paid to females, people in rural areas, and people aged over 40 for the symptomatic knee OA intervention. More nationally focused and accurate investigations of the prevalence of symptomatic knee OA are needed.

## Data Availability Statement

All datasets generated for this study are included in the article/Supplementary Material.

## Author Contributions

SL and DL conceived the study and participated in drafting the final manuscript and prepared all the figures. XX and QC analyzed the data and completed the final draft of the manuscript. All authors have read and approved the manuscript.

## Conflict of Interest

The authors declare that the research was conducted in the absence of any commercial or financial relationships that could be construed as a potential conflict of interest.

## Abbreviations

OA: osteoarthritis
BMI: body mass index
Cis: confidence intervals
AMPK: 5′-AMP-activated protein kinase.
