# Supplementary material for: The Prevalence of Symptomatic Knee Osteoarthritis in Relation to Age, Sex, Area, Region, and Body Mass Index in China: A Systematic Review and Meta-Analysis
Source: Front Med (Lausanne). 2020 Jul 16;7:304. doi: 10.3389/fmed.2020.00304 (PMC7378378; doi:10.3389/fmed.2020.00304)
Supplement: Supplementary file 1 [file Data_Sheet_1.pdf]

The prevalence of symptomatic knee osteoarthritis in relation to age, gender and region in China: a systematic review and meta-analysis

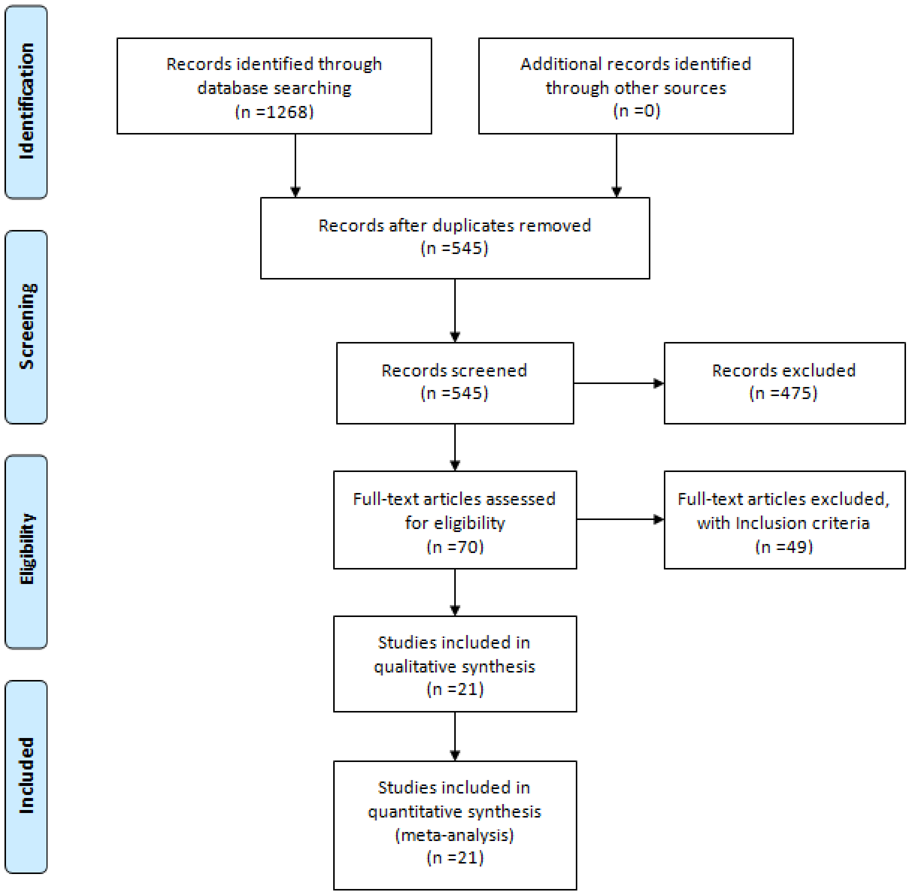

Supplementary Figure 1 Flow-chart illustrating the article search process

First, 1268 records were obtained identified through database searching. No additional records was identified through other sources. Second, 545 records left after duplicates being removed. Third, we screened the 545 records and 475 studies were excluded. Then the remaining 70 studies were full-text articles assessed for eligibility and 49 studies were excluded. Finally 21 studies were included in quantitative synthesis (meta-analysis).

Study name	Subgroup within study	Event rate	Lower limit	Upper limit	Z-Value	p-Value
Kang et al, 2009	2008<year≤2013	0.106	0.089	0.127	-21.061	0.000
Fan et al, 2010	2008<year≤2013	0.404	0.374	0.435	-6.027	0.000
Gu et al, 2011	2008<year≤2013	0.170	0.158	0.183	-34.438	0.000
Jiang et al, 2012	2008<year≤2013	0.161	0.141	0.182	-21.003	0.000
Zhang et al, 2013	2008<year≤2013	0.243	0.233	0.253	-41.140	0.000
Huang et al, 2013	2008<year≤2013	0.174	0.156	0.194	-23.127	0.000
Wang et al, 2013	2008<year≤2013	0.124	0.106	0.146	-20.912	0.000
Xiang et al, 2013	2008<year≤2013	0.333	0.310	0.357	-12.675	0.000
		0.201	0.151	0.261	-7.951	0.000
Xiang et al,2014	year>2013	0.097	0.080	0.117	-20.880	0.000
Tian et al, 2015	year>2013	0.147	0.127	0.169	-20.884	0.000
Xue et al, 2015	year>2013	0.156	0.147	0.165	-48.307	0.000
Li et al, 2015	year>2013	0.203	0.187	0.220	-26.382	0.000
Tang et al, 2016	year>2013	0.081	0.077	0.085	-86.727	0.000
Liu et al, 2016	year>2013	0.166	0.154	0.179	-35.188	0.000
Zhang et al, 2016	year>2013	0.242	0.221	0.265	-18.672	0.000
		0.149	0.106	0.204	-8.905	0.000
Du et al, 2005	year≤2008	0.072	0.062	0.084	-30.231	0.000
Zang et al, 2006	year≤2008	0.121	0.111	0.132	-40.460	0.000
Zeng et al, 2006	year≤2008	0.109	0.097	0.123	-30.627	0.000
Tang et al , 2007	year≤2008	0.043	0.039	0.046	-71.969	0.000
Wang et al, 2007	year≤2008	0.095	0.078	0.116	-20.079	0.000
Wang et al , 2008	year≤2008	0.140	0.126	0.155	-29.778	0.000
		0.091	0.058	0.139	-9.419	0.000

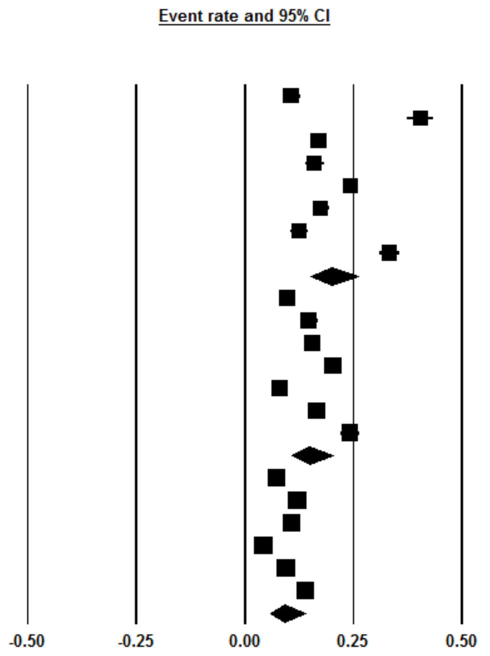

Supplementary Figure 2 Forest plot of the prevalence rates of symptomatic knee osteoarthritis by study year. CI=confidence interval.

A total of 21 studies were included in this subgroup meta-analysis. The prevalence of symptomatic knee OA presented a rapid growth trend from period before 2008 (9.1%, 95% CI=5.8-13.9%) to period between 2008 and 2013(20.1%, 95% CI=15.1-26.1%); however, the prevalence after 2013 dropped to 14.9% (95% CI=10.6-20.4%).

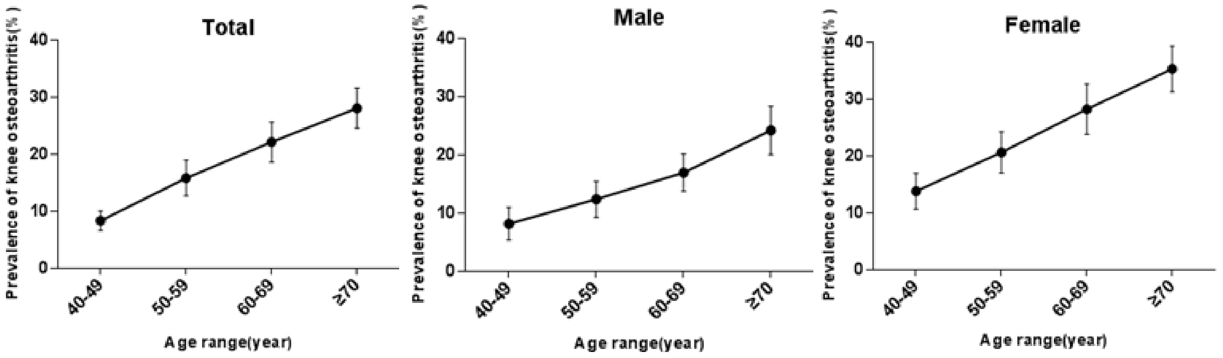

Supplementary Figure 3 Line chart of the prevalence rates of symptomatic knee osteoarthritis by age

The prevalence rates of symptomatic knee osteoarthritis increased with age in both sexes and almost showed a linear growth after 40 years old. Females showed a more impressive growth compared with males.

Study name	Subgroup within study	Event rate	Lower limit	Upper limit	Z-Value	p-Value
		Statistics for each study				
Zhang et al, 2013	≥70	0.481	0.470	0.493	-3.140	0.002
Zang et al, 2006	≥70	0.218	0.205	0.231	-33.009	0.000
Tian et al, 2015	≥70	0.187	0.165	0.211	-19.220	0.000
Wang et al , 2008	≥70	0.188	0.172	0.205	-27.018	0.000
Tang et al, 2016	≥70	0.059	0.056	0.063	-85.401	0.000
Jiang et al, 2012	≥70	0.258	0.234	0.284	-15.979	0.000
Xiang et al, 2013	≥70	0.390	0.366	0.415	-8.447	0.000
Liu et al, 2016	≥70	0.247	0.233	0.262	-28.136	0.000
Kang et al, 2009	≥70	0.180	0.158	0.205	-18.697	0.000
Xiang et al,2014	≥70	0.101	0.083	0.121	-20.838	0.000
Gu et al, 2011	≥70	0.140	0.129	0.152	-36.407	0.000
Fan et al, 2010	≥70	0.432	0.401	0.462	-4.323	0.000
		0.216	0.134	0.329	-4.393	0.000
Zhang et al, 2013	15-29	0.029	0.026	0.033	-49.833	0.000
Zang et al, 2006	15-29	0.002	0.001	0.003	-16.095	0.000
		0.007	0.000	0.113	-3.367	0.001
Zhang et al, 2013	30-39	0.115	0.108	0.122	-54.973	0.000
Zang et al, 2006	30-39	0.023	0.019	0.028	-35.163	0.000
		0.052	0.010	0.228	-3.393	0.001
Zhang et al, 2013	40-49	0.265	0.255	0.276	-37.977	0.000
Zang et al, 2006	40-49	0.050	0.044	0.057	-40.171	0.000
Tian et al, 2015	40-49	0.018	0.012	0.028	-17.834	0.000
Wang et al , 2008	40-49	0.025	0.019	0.032	-27.040	0.000
Tang et al, 2016	40-49	0.033	0.030	0.036	-78.967	0.000
Jiang et al, 2012	40-49	0.055	0.044	0.070	-22.438	0.000
Xiang et al, 2013	40-49	0.065	0.054	0.079	-25.448	0.000
Zhang et al, 2016	40-49	0.084	0.070	0.099	-25.332	0.000
Liu et al, 2016	40-49	0.091	0.082	0.101	-38.757	0.000
		0.058	0.026	0.121	-6.741	0.000
Zhang et al, 2013	50-59	0.381	0.370	0.393	-19.846	0.000
Zang et al, 2006	50-59	0.109	0.100	0.119	-40.957	0.000
Tian et al, 2015	50-59	0.030	0.021	0.042	-19.889	0.000
Wang et al , 2008	50-59	0.038	0.031	0.047	-29.209	0.000
Tang et al, 2016	50-59	0.057	0.054	0.061	-85.141	0.000
Jiang et al, 2012	50-59	0.126	0.108	0.146	-22.236	0.000
Xiang et al, 2013	50-59	0.162	0.144	0.182	-23.444	0.000
Zhang et al, 2016	50-59	0.160	0.142	0.179	-23.255	0.000
Liu et al, 2016	50-59	0.193	0.180	0.207	-33.040	0.000
Kang et al, 2009	50-59	0.030	0.021	0.042	-19.031	0.000
Xiang et al,2014	50-59	0.077	0.062	0.096	-20.942	0.000
		0.096	0.052	0.170	-6.680	0.000
Zhang et al, 2013	60-69	0.418	0.407	0.429	-13.781	0.000
Zang et al, 2006	60-69	0.141	0.131	0.153	-39.324	0.000
Tian et al, 2015	60-69	0.099	0.083	0.118	-22.122	0.000
Wang et al , 2008	60-69	0.085	0.074	0.097	-31.330	0.000
Tang et al, 2016	60-69	0.075	0.071	0.079	-86.602	0.000
Jiang et al, 2012	60-69	0.256	0.232	0.281	-16.114	0.000
Xiang et al, 2013	60-69	0.269	0.247	0.292	-17.164	0.000
Liu et al, 2016	60-69	0.200	0.187	0.214	-32.432	0.000
Kang et al, 2009	60-69	0.130	0.111	0.152	-20.517	0.000
Xiang et al,2014	60-69	0.056	0.043	0.072	-20.531	0.000
Gu et al, 2011	60-69	0.053	0.046	0.061	-37.352	0.000
Fan et al, 2010	60-69	0.253	0.227	0.281	-14.883	0.000
		0.144	0.087	0.230	-6.101	0.000

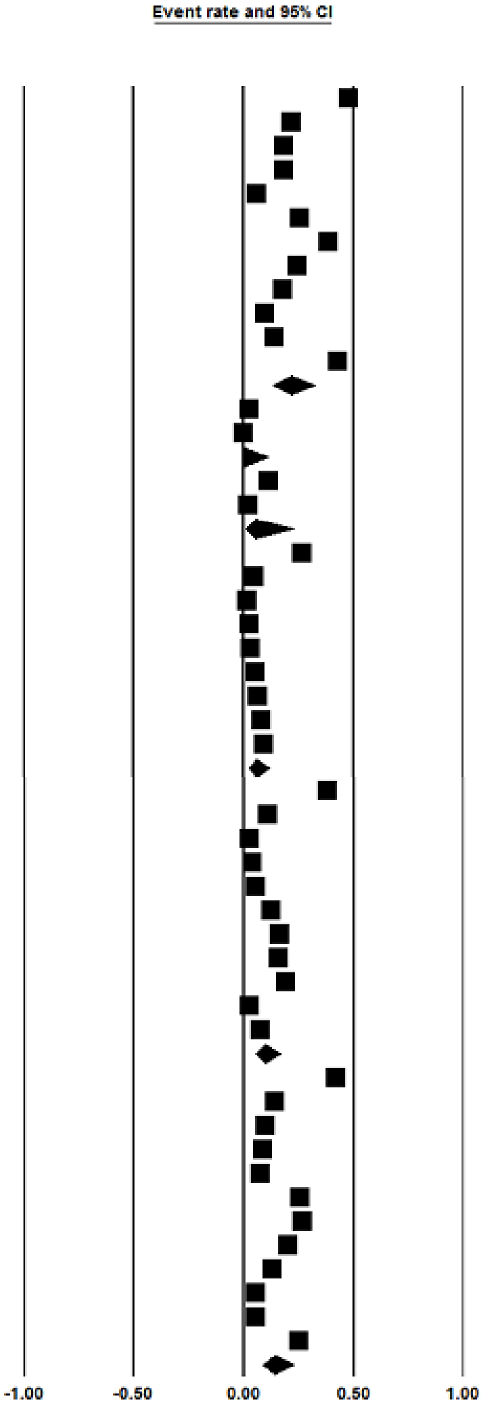

Supplementary Figure 4 Forest plot of the prevalence rates of symptomatic knee osteoarthritis by age in men. CI=confidence interval.

The different prevalence of symptomatic knee osteoarthritis with age in men was evaluated as follows: 0.7% (95% CI=0.0-11.3%) in 15- to 29-year age group, 5.2% (95% CI=1.0-22.8%) in 30- to 39-year age group, 5.8% (95% CI=2.6-12.1%) in 40- to 49-year age group, 9.6% (95% CI=5.2-17.0%) in 50- to 59-year age group, 14.4% (95% CI=8.7-23.0%) in 60- to 69-year age group, 21.6% (95% CI=13.4-32.9%) in the group over 70 years of age.

Study name	Subgroup within study	Event rate	Lower limit	Upper limit	Z-Value	p-Value
Zhang et al, 2013	≥70	0.574	0.563	0.586	12.464	0.000
Zang et al, 2006	≥70	0.332	0.318	0.347	-20.590	0.000
Tian et al, 2015	≥70	0.413	0.385	0.442	-5.806	0.000
Wang et al , 2008	≥70	0.297	0.278	0.316	-18.638	0.000
Tang et al, 2016	≥70	0.115	0.110	0.120	-85.201	0.000
Liu et al, 2016	≥70	0.293	0.278	0.308	-23.526	0.000
Jiang et al, 2012	≥70	0.412	0.384	0.440	-6.068	0.000
Xiang et al, 2013	≥70	0.514	0.489	0.539	1.084	0.278
Xiang et al,2014	≥70	0.191	0.167	0.216	-17.966	0.000
Kang et al, 2009	≥70	0.310	0.282	0.339	-11.876	0.000
Gu et al, 2011	≥70	0.262	0.248	0.277	-26.300	0.000
Fan et al, 2010	≥70	0.526	0.495	0.557	1.664	0.096
		0.318	0.313	0.323	-66.993	0.000
Zhang et al, 2013	15-29	0.028	0.025	0.032	-49.433	0.000
		0.028	0.025	0.032	-49.433	0.000
Zhang et al, 2013	30-39	0.132	0.124	0.140	-53.846	0.000
Zang et al, 2006	30-39	0.033	0.028	0.039	-37.692	0.000
		0.111	0.104	0.117	-63.861	0.000
Zhang et al, 2013	40-49	0.281	0.271	0.292	-35.589	0.000
Zang et al, 2006	40-49	0.126	0.116	0.136	-40.231	0.000
Tian et al, 2015	40-49	0.052	0.040	0.067	-21.620	0.000
Wang et al , 2008	40-49	0.064	0.055	0.075	-31.042	0.000
Tang et al, 2016	40-49	0.068	0.064	0.072	-86.249	0.000
Liu et al, 2016	40-49	0.096	0.086	0.106	-38.680	0.000
Jiang et al, 2012	40-49	0.108	0.091	0.126	-22.673	0.000
Xiang et al, 2013	40-49	0.108	0.093	0.125	-25.372	0.000
Zhang et al, 2016	40-49	0.272	0.250	0.295	-16.752	0.000
		0.146	0.142	0.150	-111.536	0.000
Zhang et al, 2013	50-59	0.410	0.398	0.421	-15.194	0.000
Zang et al, 2006	50-59	0.269	0.255	0.283	-27.727	0.000
Tian et al, 2015	50-59	0.097	0.081	0.116	-22.147	0.000
Wang et al , 2008	50-59	0.158	0.143	0.174	-28.851	0.000
Tang et al, 2016	50-59	0.091	0.087	0.095	-86.629	0.000
Liu et al, 2016	50-59	0.173	0.160	0.186	-34.686	0.000
Jiang et al, 2012	50-59	0.239	0.216	0.264	-17.082	0.000
Xiang et al, 2013	50-59	0.350	0.326	0.375	-11.432	0.000
Zhang et al, 2016	50-59	0.328	0.305	0.353	-12.850	0.000
Xiang et al,2014	50-59	0.066	0.052	0.083	-20.801	0.000
Kang et al, 2009	50-59	0.090	0.074	0.109	-21.250	0.000
		0.225	0.221	0.230	-94.331	0.000
Zhang et al, 2013	60-69	0.507	0.495	0.519	1.182	0.237
Zang et al, 2006	60-69	0.388	0.373	0.404	-13.859	0.000
Tian et al, 2015	60-69	0.225	0.202	0.250	-17.322	0.000
Wang et al , 2008	60-69	0.200	0.184	0.217	-26.215	0.000
Tang et al, 2016	60-69	0.147	0.142	0.152	-81.487	0.000
Liu et al, 2016	60-69	0.215	0.201	0.229	-31.195	0.000
Jiang et al, 2012	60-69	0.378	0.351	0.406	-8.365	0.000
Xiang et al, 2013	60-69	0.524	0.499	0.549	1.858	0.063
Xiang et al,2014	60-69	0.079	0.064	0.098	-20.951	0.000
Kang et al, 2009	60-69	0.290	0.263	0.318	-13.039	0.000
Gu et al, 2011	60-69	0.066	0.058	0.075	-38.036	0.000
Fan et al, 2010	60-69	0.371	0.342	0.402	-8.048	0.000
		0.285	0.280	0.290	-78.967	0.000

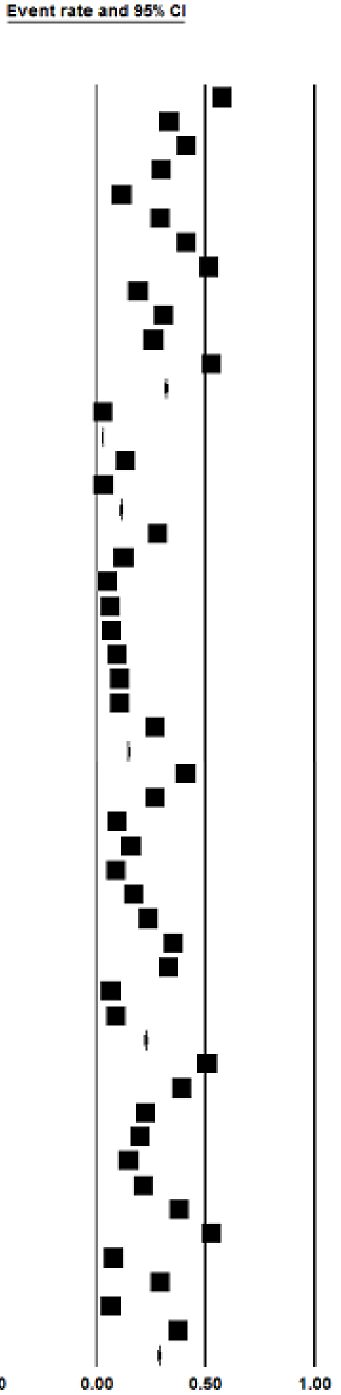

Supplementary Figure 5 Forest plot of the prevalence rates of symptomatic knee osteoarthritis by age in women. CI=confidence interval.

The different prevalence rates of symptomatic knee osteoarthritis with age in males was evaluated as follows: 2.8% (95% CI=2.5-3.2%) in 15- to 29-year age group, 11.1% (95% CI=10.4-11.7%) in 30- to 39-year age group, 14.6% (95% CI=14.2-15.0%) in 40- to 49-year age group, 22.5% (95% CI=22.1-23.0%) in 50- to 59-year age group, 28.5% (95% CI=28.0-29.0%) in 60- to 69-year age group, 31.8% (95% CI=31.3-32.3%) in the group over 70 years of age.

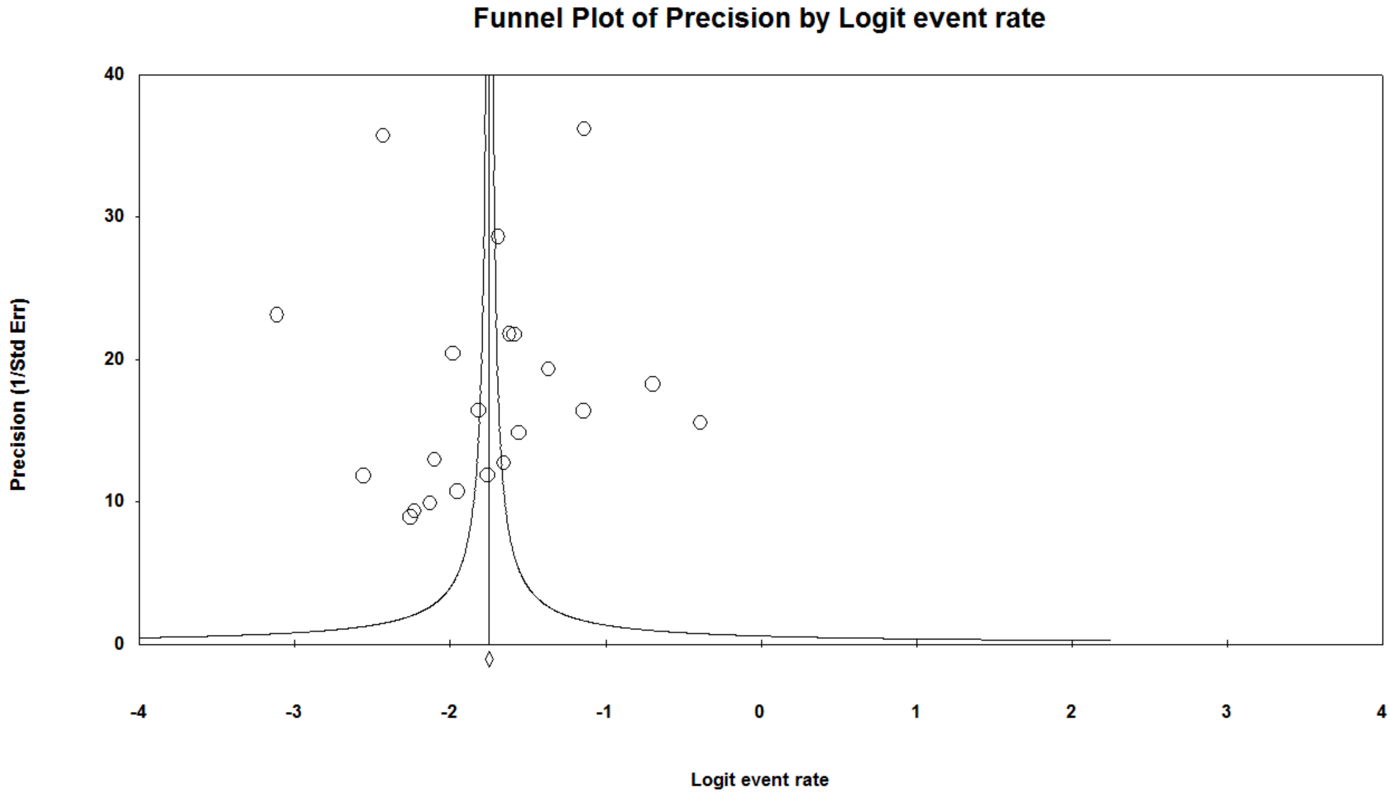

Supplementary Figure 6 Funnel plots for the overall pooled studies.

The total studies arrange around the center line symmetrically, so there was no publication bia.

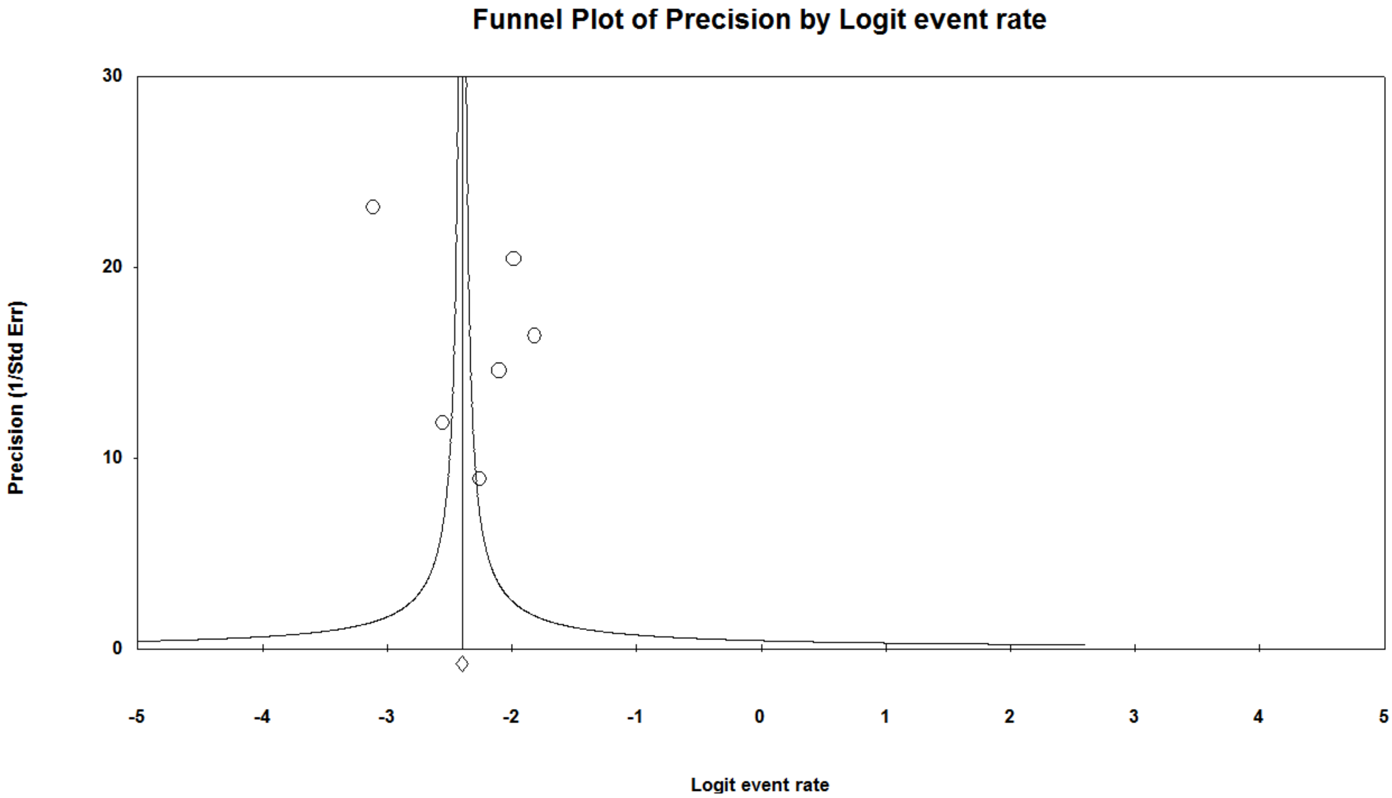

Supplementary Figure 7 Funnel plots for the subgroup of study year between 1990 and 2008.

A total of 6 studies surveyed the prevalence of symptomatic knee osteoarthritis between 1990 and 2008. The 6 studies arrange around the center line symmetrically, so there was no publication bia.

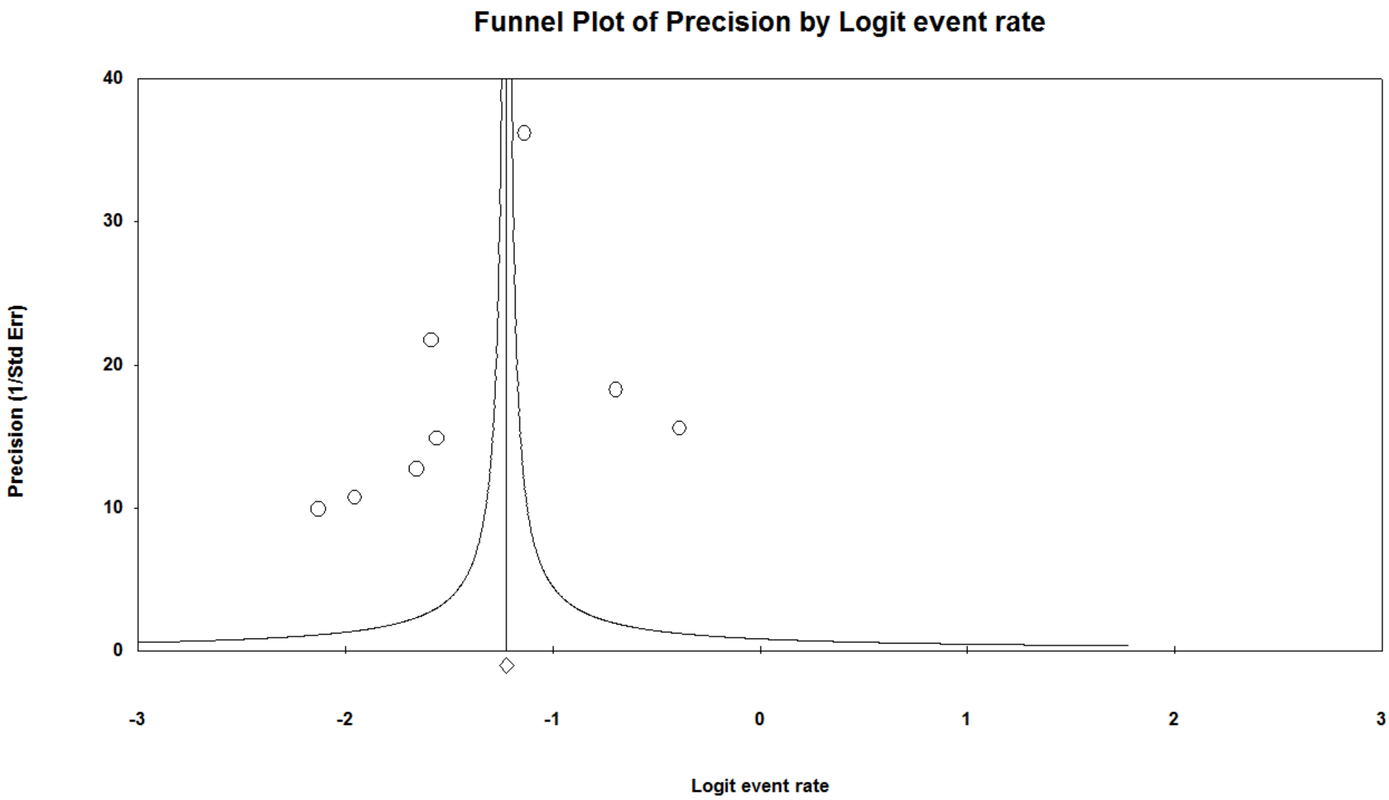

Supplementary Figure 8 Funnel plots for the subgroup of study year between 2008 and 2013.
A total of 8 studies surveyed the prevalence of symptomatic knee osteoarthritis between 2008 and 2013. The 8 studies arrange around the center line symmetrically, so there was no publication bia.

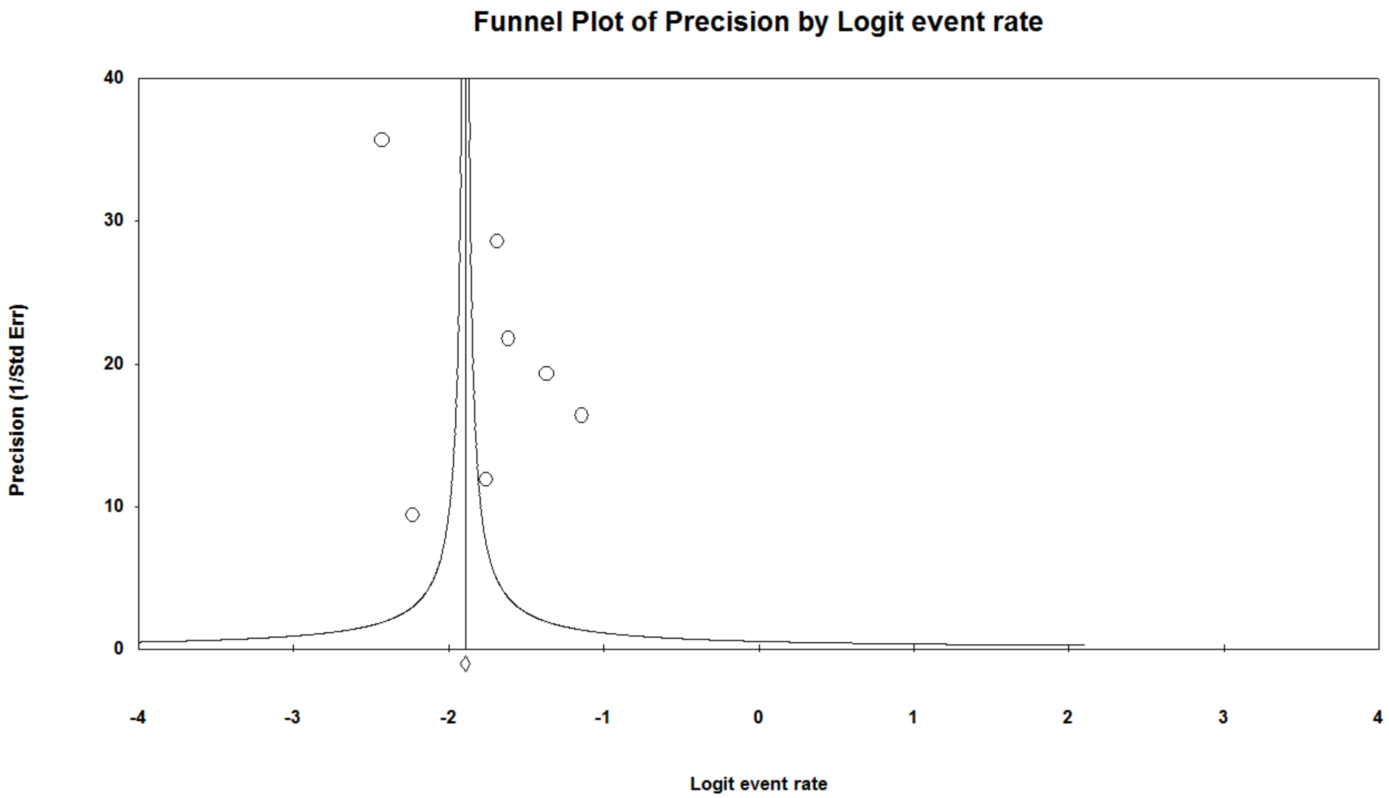

Supplementary Figure 9 Funnel plots for the subgroup of study year after 2013.
A total of 7 studies surveyed the prevalence of symptomatic knee osteoarthritis after 2013. The 7 studies arrange around the center line symmetrically, so there was no publication bia.

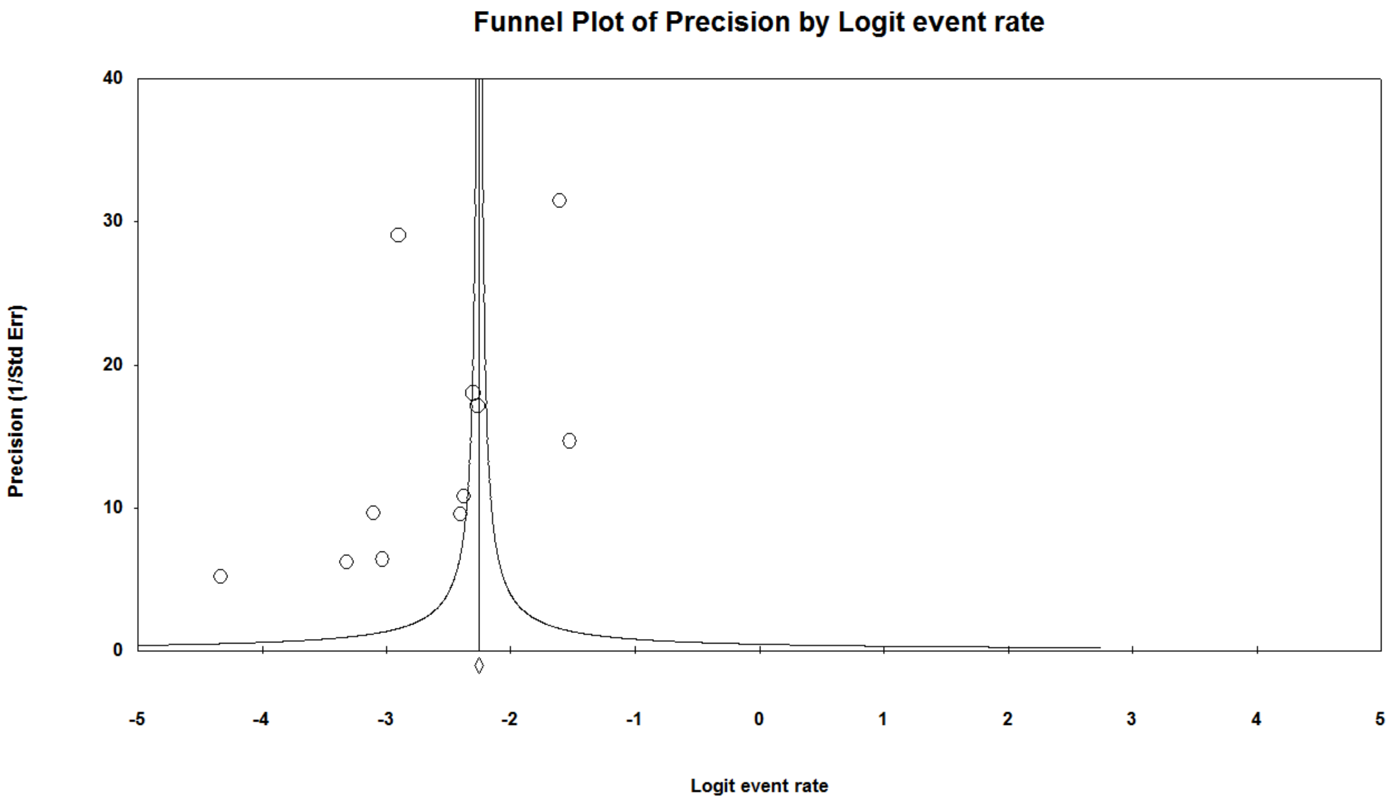

Supplementary Figure 10 Funnel plots for the 40-49 year age group.
A total of 11 studies investigated the prevalence of symptomatic knee osteoarthritis in age group of 40-49 years. The studies arrange around the center line symmetrically, so there was no publication bia.

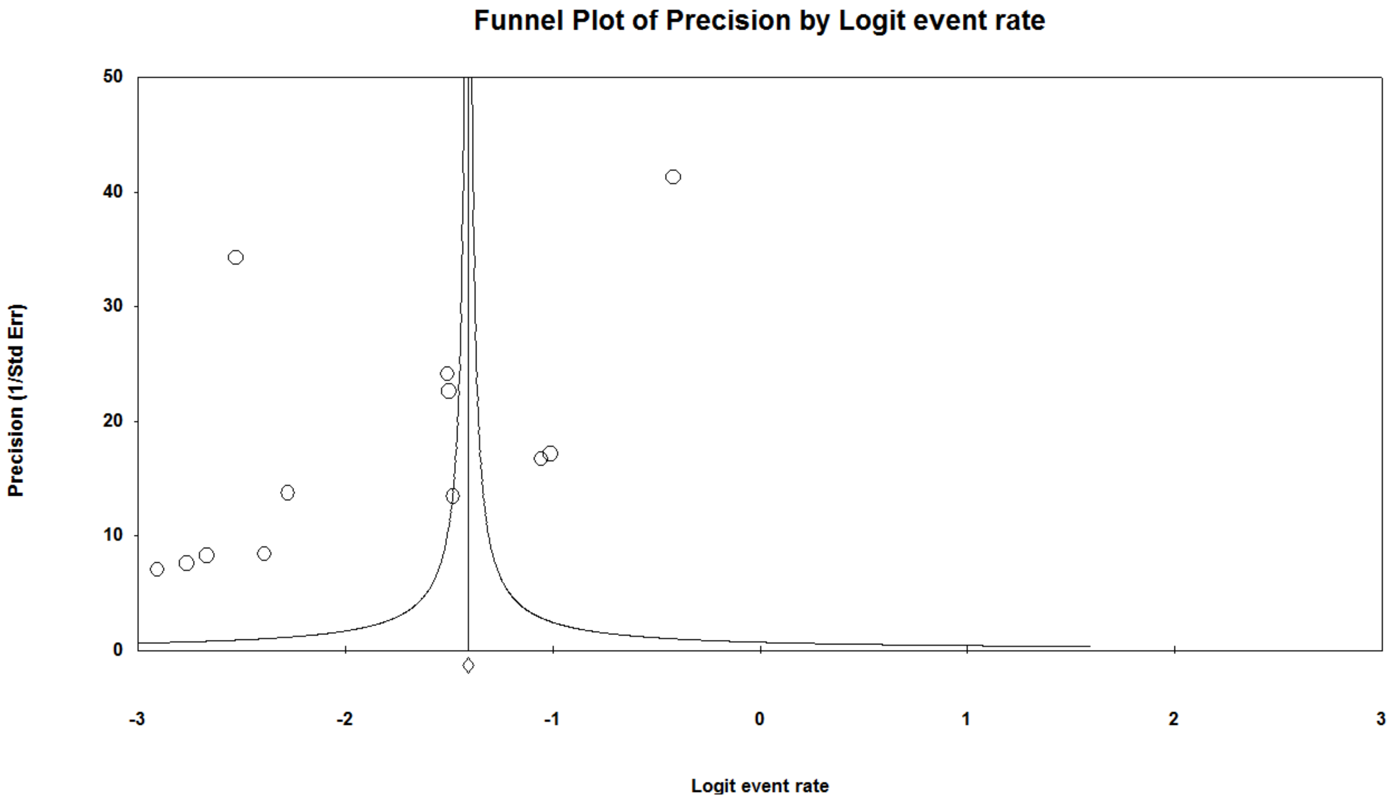

Supplementary Figure 11 Funnel plots for the 50-59 year age group.
A total of 12 studies investigated the prevalence of symptomatic knee osteoarthritis in age group of 50-59 years. The studies arrange around the center line symmetrically, so there was no publication bia.

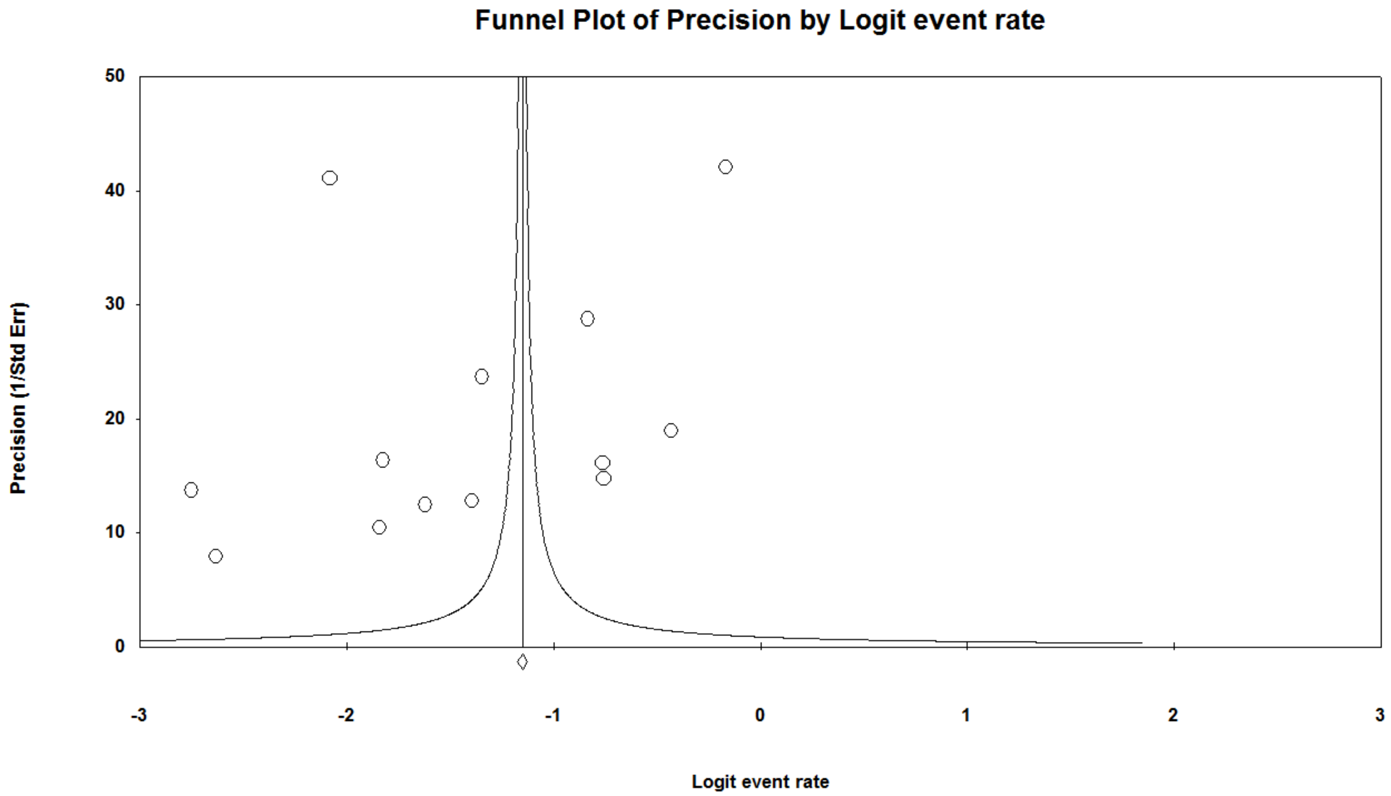

Supplementary Figure 12 Funnel plots for the 60-69 year age group.

A total of 13 studies investigated the prevalence of symptomatic knee osteoarthritis in age group of 60-69 years. The studies arrange around the center line symmetrically, so there was no publication bia.

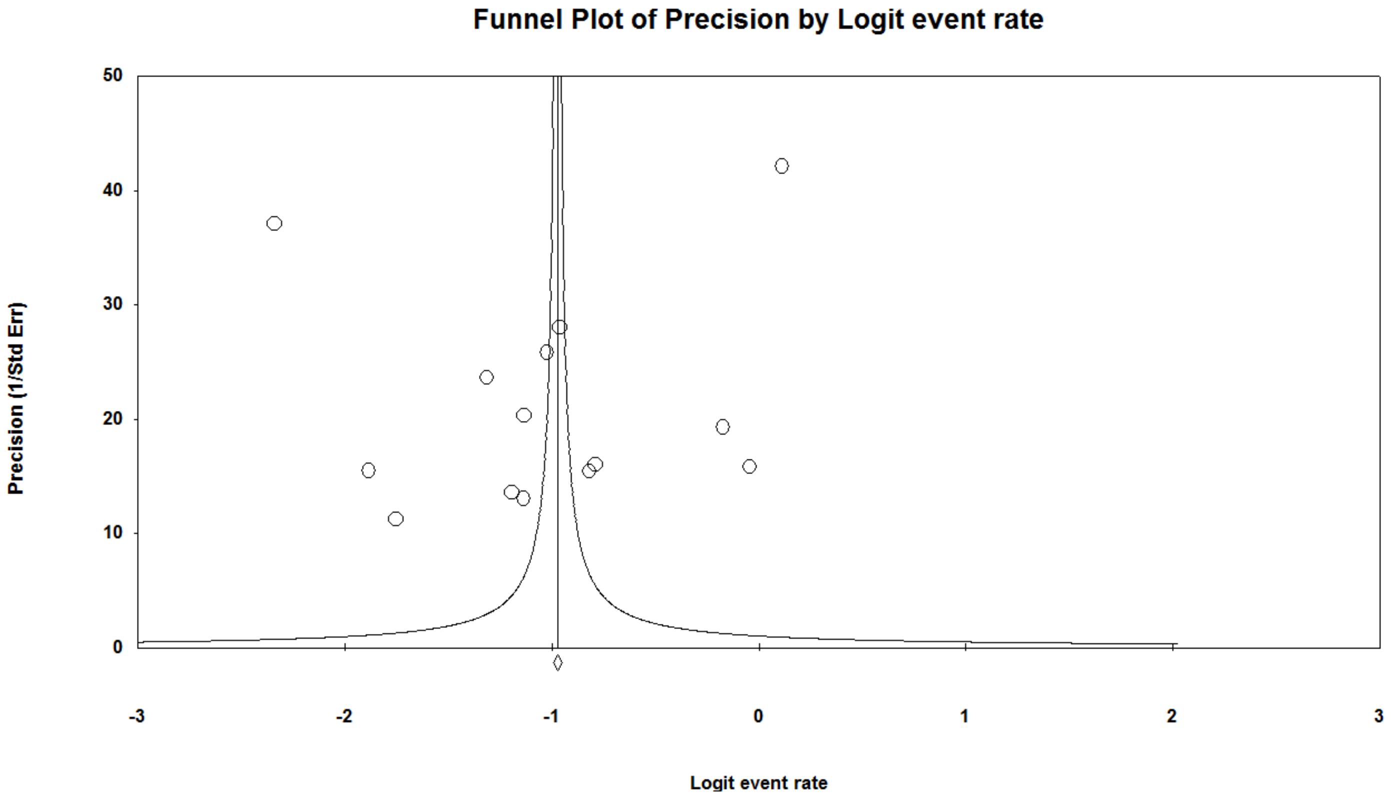

Supplementary Figure 13 Funnel plots for the group over 70 years of age.

A total of 14 studies investigated the prevalence of symptomatic knee osteoarthritis in the group over 70 years of age. The 14 studies arrange around the center line symmetrically, so there was no publication bia.

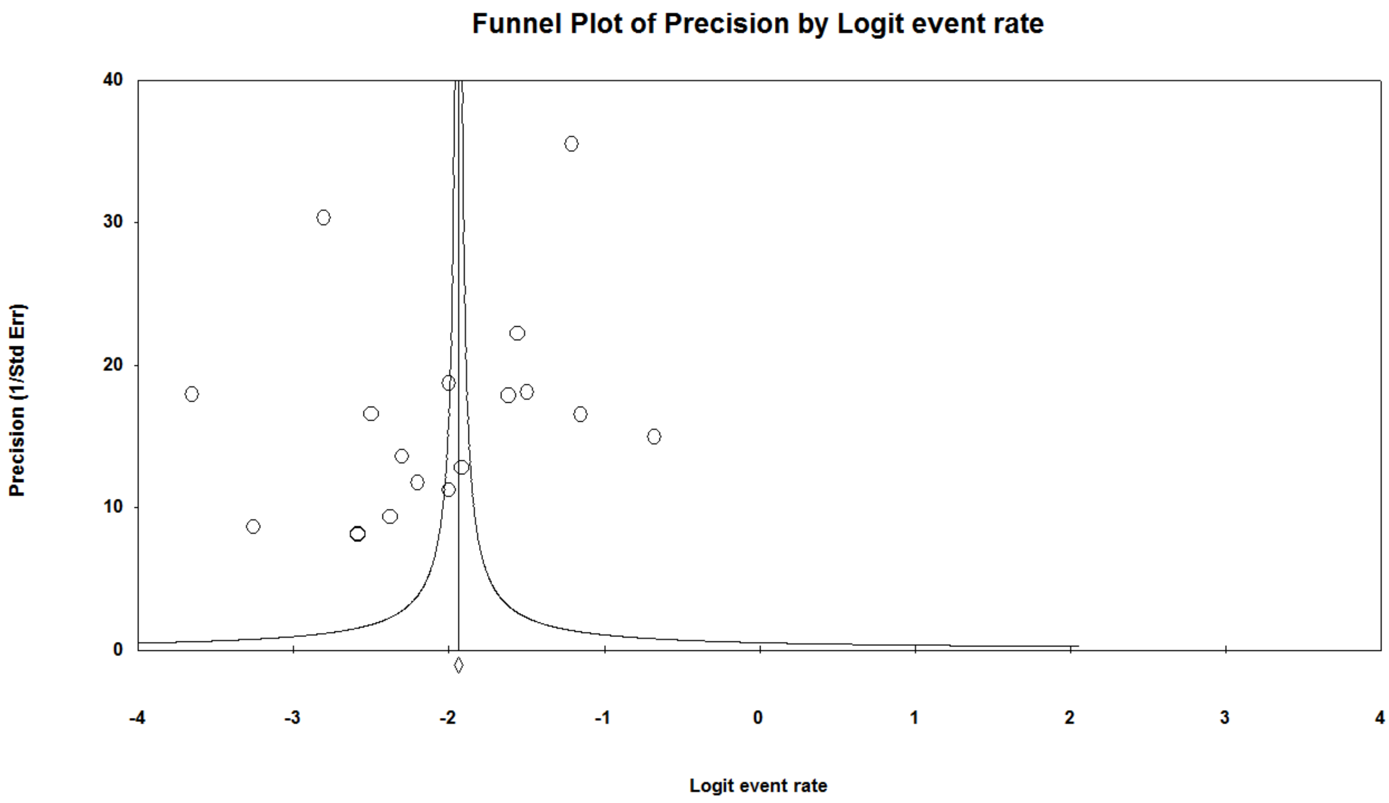

Supplementary Figure 14 Funnel plots for the male subgroup.
A total of 18 studies investigated the prevalence of symptomatic knee osteoarthritis in men. The 18 studies arrange around the center line symmetrically, so there was no publication bia.

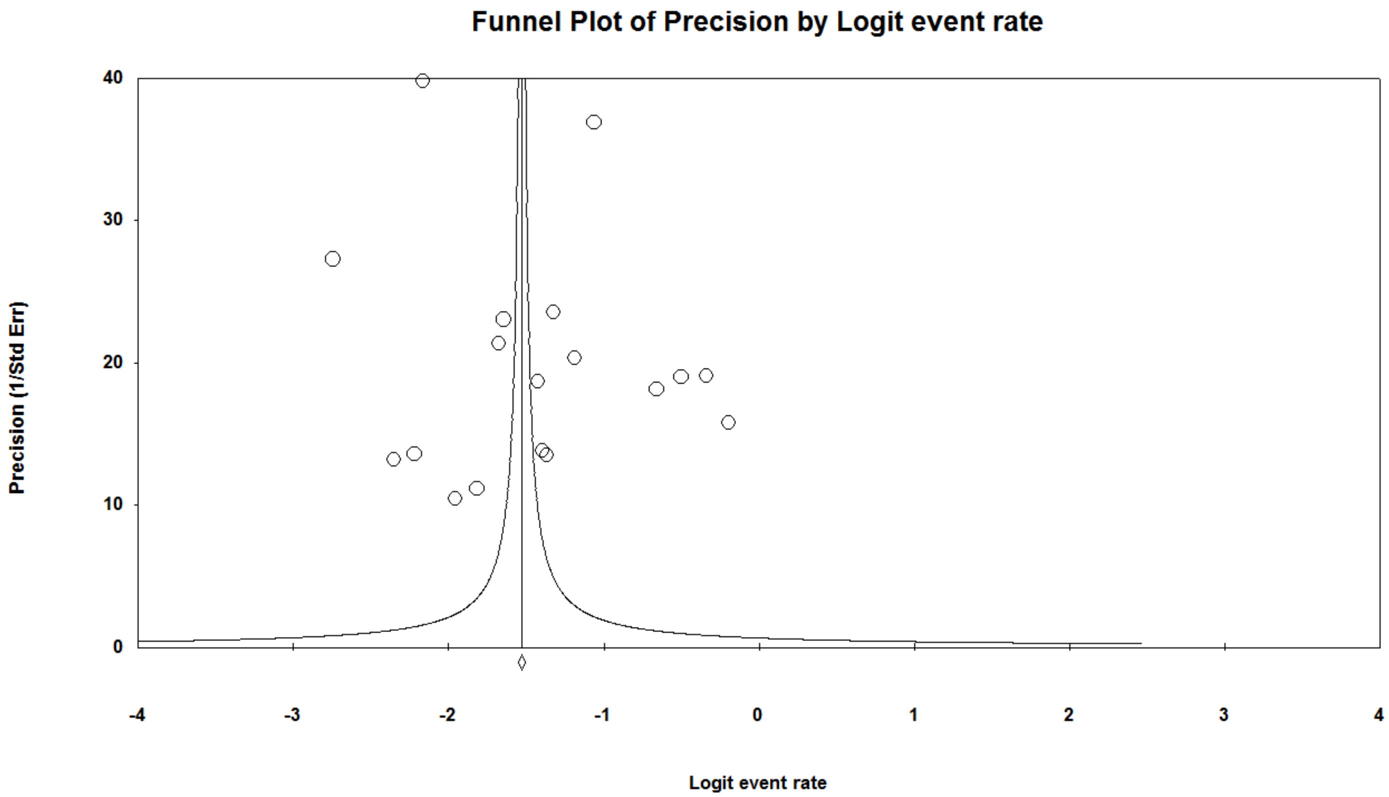

Supplementary Figure 15 Funnel plots for the female subgroup.
A total of 18 studies investigated the prevalence of symptomatic knee osteoarthritis in women. The 18 studies arrange around the center line symmetrically, so there was no publication bia.

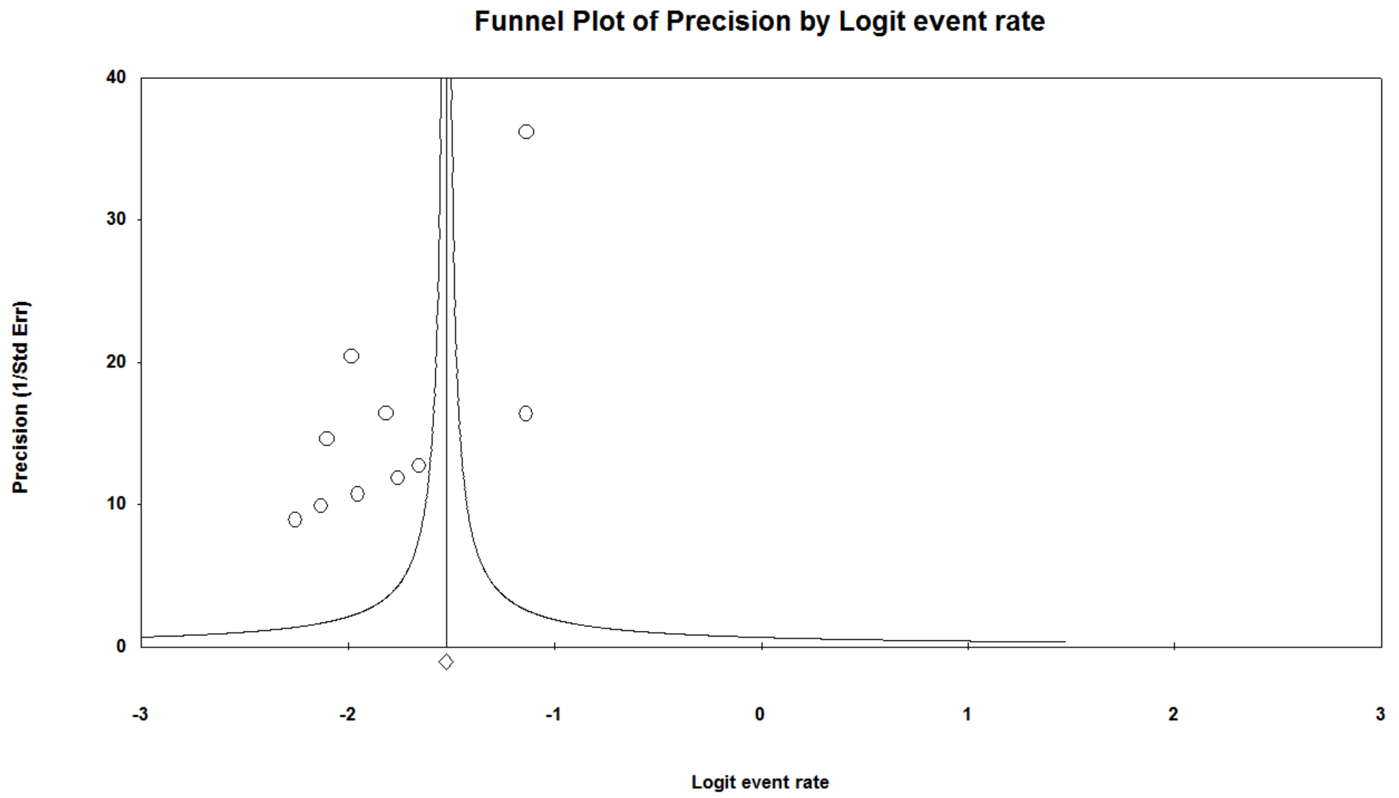

Supplementary Figure 16　Funnel plots for the Northern subgroup.

A total of 10 studies investigated the prevalence of symptomatic knee osteoarthritis in Northern China. The 10 studies arrange around the center line symmetrically, so there was no publication bia.

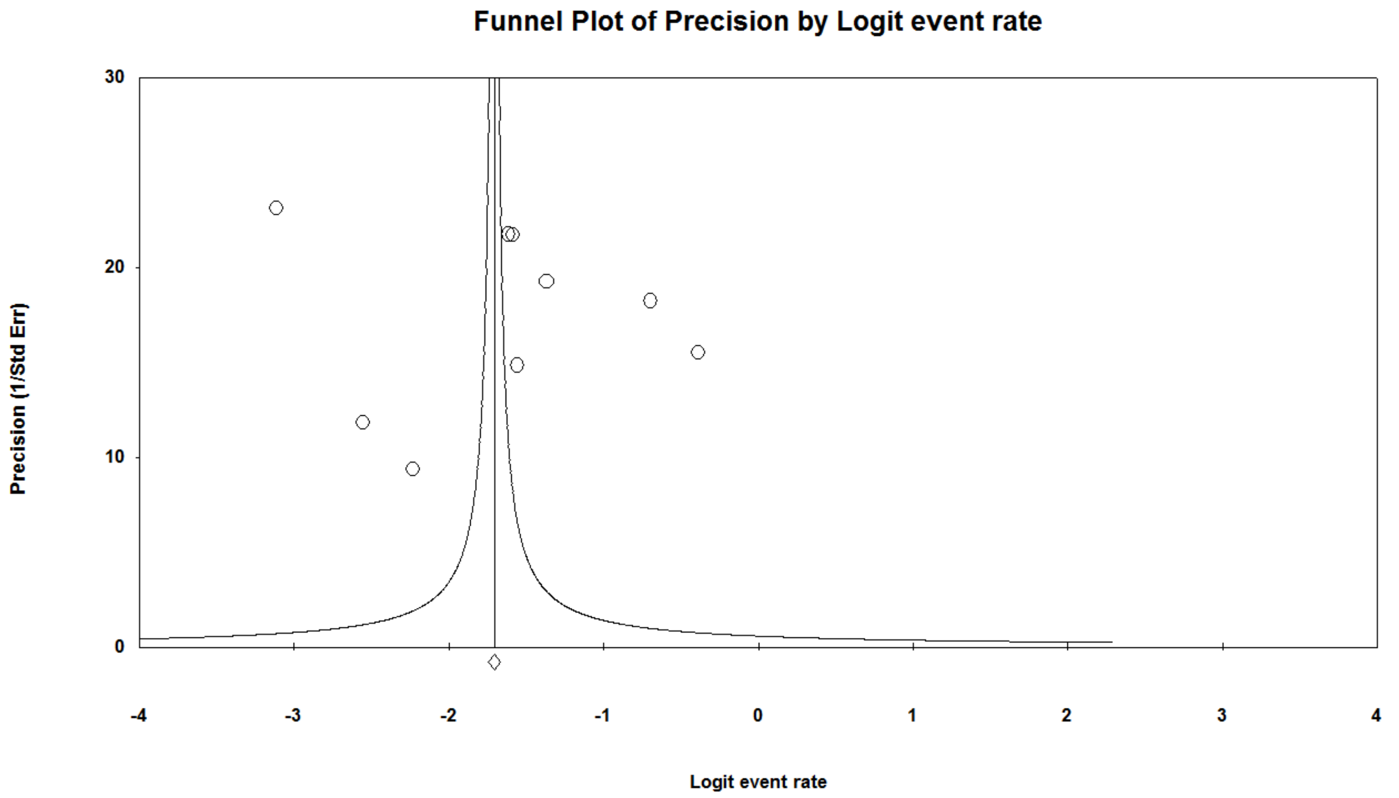

Supplementary Figure 17　Funnel plots for the Southern subgroup.

A total of 9 studies investigated the prevalence of symptomatic knee osteoarthritis in Southern China. The 9 studies arrange around the center line symmetrically, so there was no publication bia.

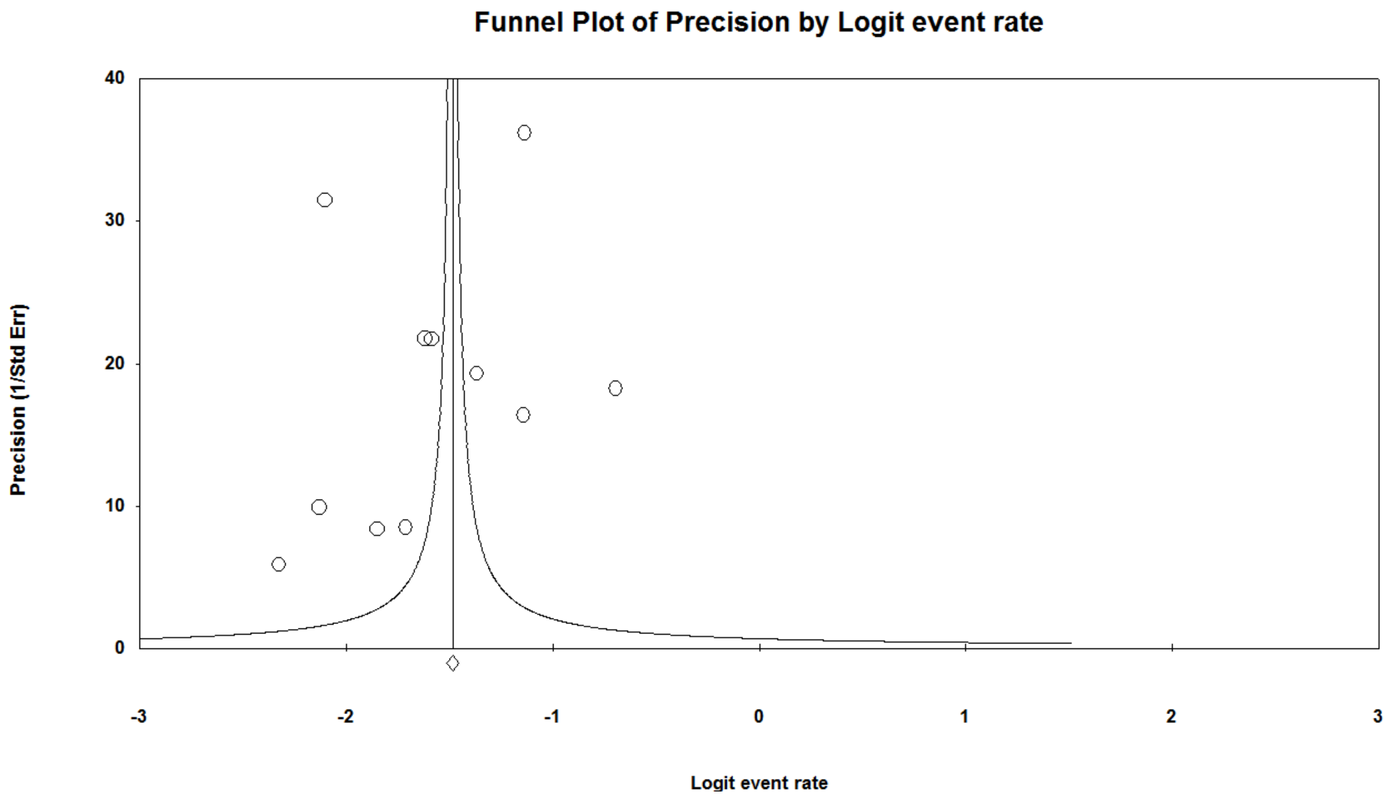

Supplementary Figure 18 Funnel plots for the rural subgroup.

A total of 11 studies investigated the prevalence of symptomatic knee osteoarthritis in rural population. The 11 studies arrange around the center line symmetrically, so there was no publication bia.

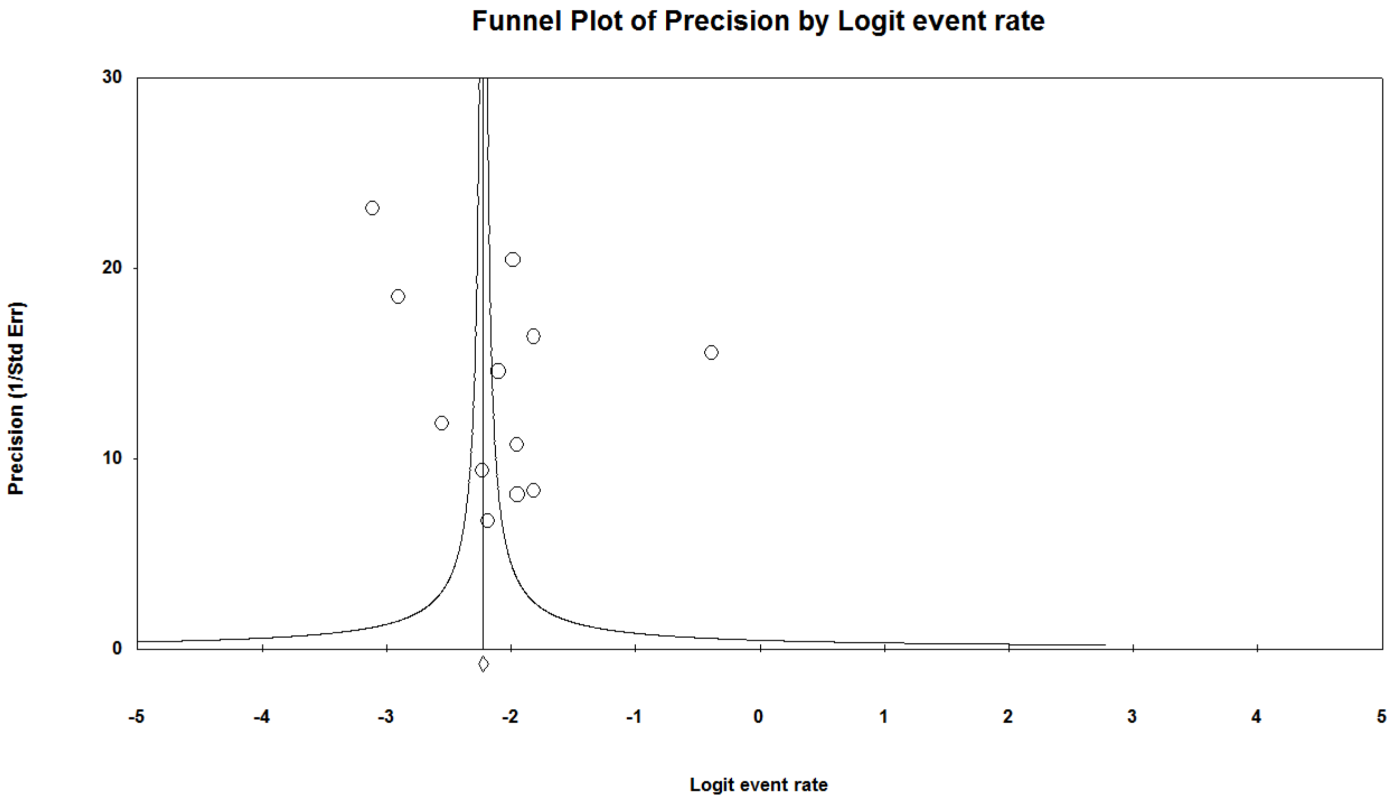

Supplementary Figure 19 Funnel plots for the ubran subgroup.

A total of 12 studies investigated the prevalence of symptomatic knee osteoarthritis in ubran population. The 12 studies arrange around the center line symmetrically, so there was no publication bia.

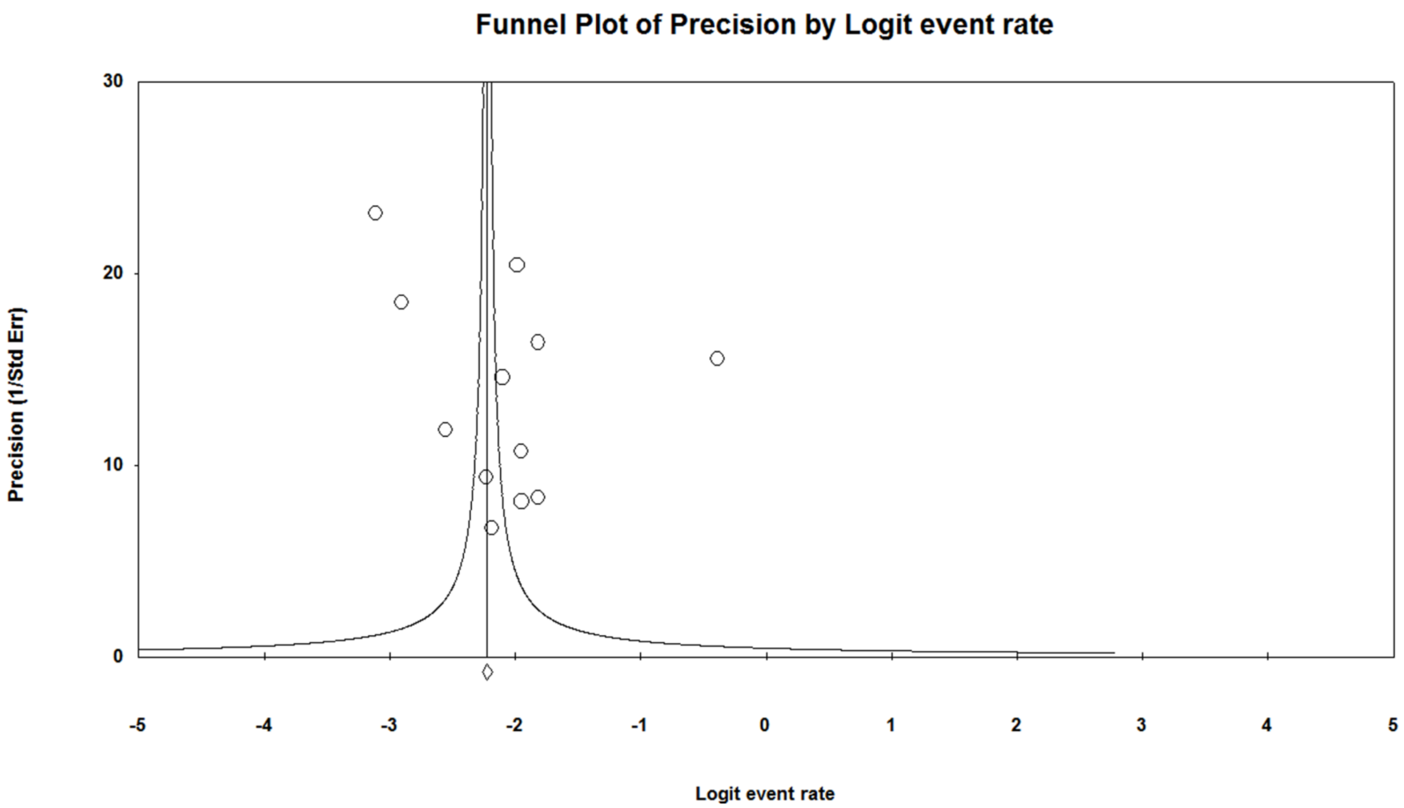

Supplementary Figure 20 Funnel plots for the ubran subgroup.

A total of 12 studies investigated the prevalence of symptomatic knee osteoarthritis in ubran population. The 12 studies arrange around the center line symmetrically, so there was no publication bias.

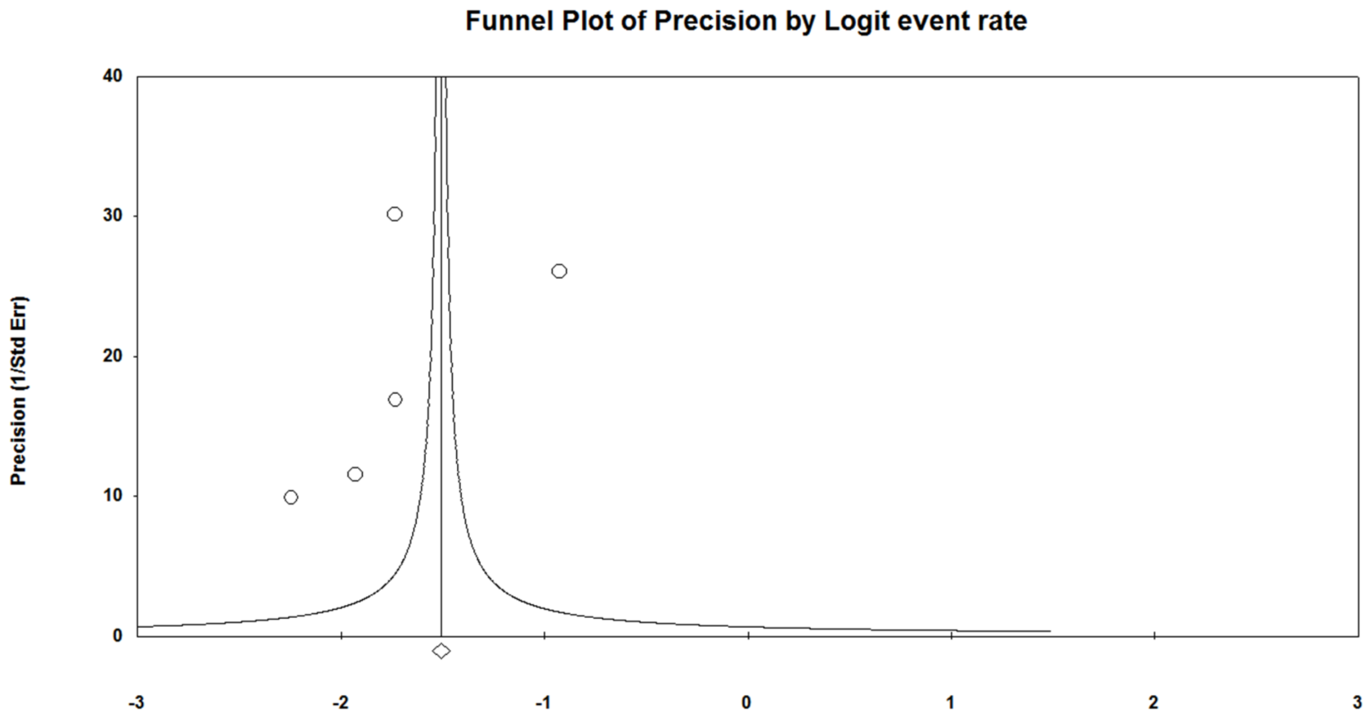

Supplementary Figure 21 Funnel plots for the subgroup of BMI≤24.

A total of 5 studies investigated the prevalence of symptomatic knee osteoarthritis in BMI≤24 subgroup. The 5 studies arrange around the center line symmetrically, so there was no publication bias.

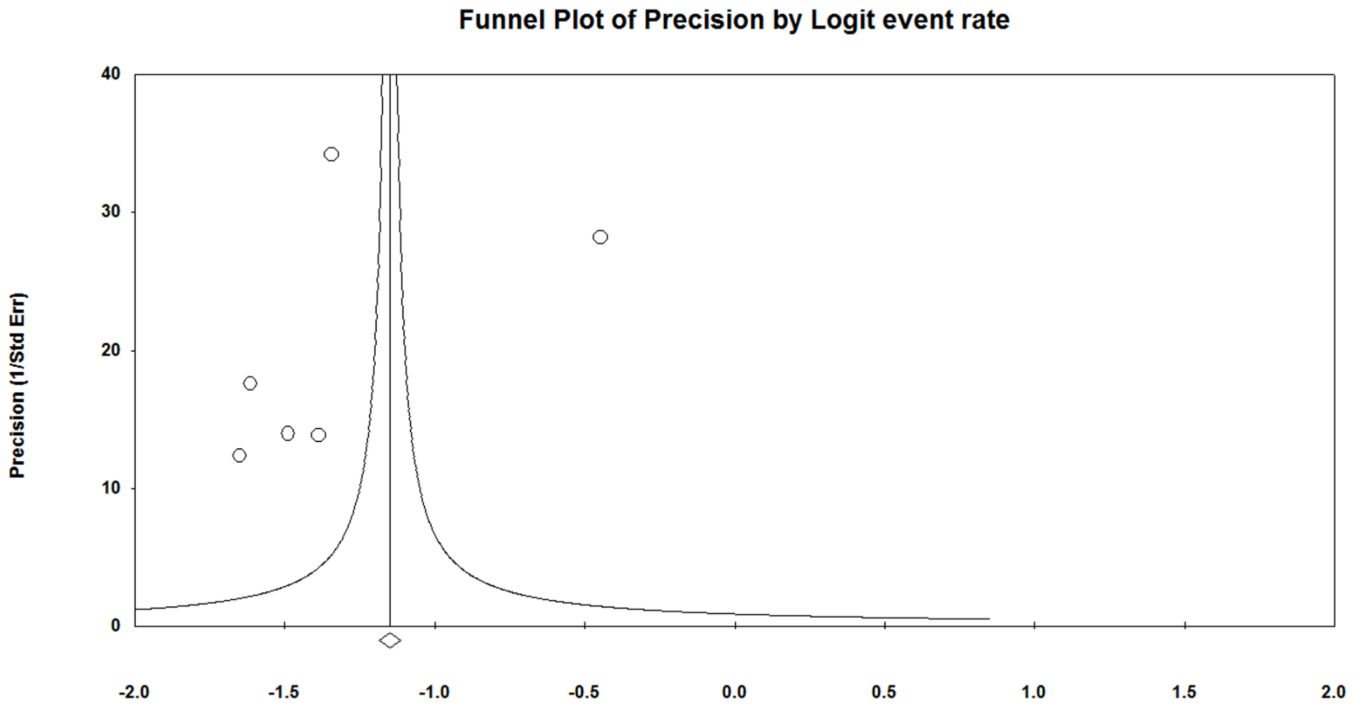

Supplementary Figure 22 Funnel plots for the subgroup of 24＜BMI＜28.

A total of 6 studies investigated the prevalence of symptomatic knee osteoarthritis in 24＜BMI＜28 subgroup. The 6 studies arrange around the center line symmetrically, so there was no publication bias.

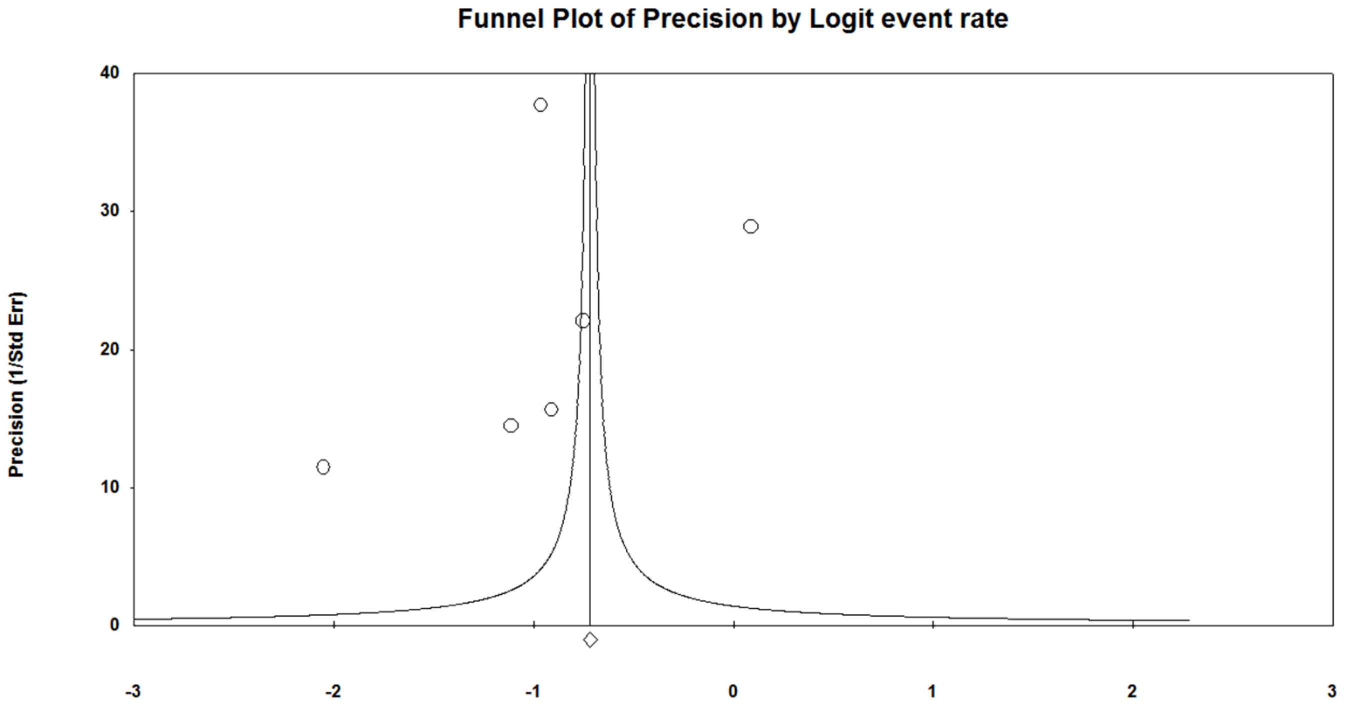

Supplementary Figure 23 Funnel plots for the subgroup of BMI≥28.

A total of 6 studies investigated the prevalence of symptomatic knee osteoarthritis in BMI≥28 subgroup. The 6 studies arrange around the center line symmetrically, so there was no publication bias.

Supplementary table 1. Details of electronic bibliographic database search strategies

**Database**	**Search strategies**
Pubmed	((("osteoarthritis"[MeSH Terms] AND (("prevalence"[MeSH Terms]) OR "incidence"[MeSH Terms] OR "epidemiology"[MeSH Terms])) AND ("China"[MeSH Terms]) OR "Chinese"[MeSH Terms])
Embase	Title or Abstract osteoarthritis AND Title or Abstract (China or Chinese) AND Title or Abstract (prevalence or incidence or epidemiology)
Web of science	Title or Abstract:(osteoarthritis) AND Title or Abstract: (prevalence or incidence or epidemiology) AND Title or Abstract: (China or Chinese)
China National Knowledge Infrastructure Periodicals	Keywords: osteoarthritis AND Keywords: prevalence or incidence or epidemiology
The VIP Database for Chinese Technical	(1) Title or Keywords: osteoarthritis; Property: dim AND Title or Keywords: prevalence; Property: dim<br>(2) Title or Keywords: osteoarthritis; Property: dim AND Title or Keywords: incidence; Property: dim<br>(3) Title or Keywords: osteoarthritis; Property: dim AND Title or Keywords: epidemiology; Property: dim
The Wan Fang Database for Chinese Periodicals	(1) Title or Keywords: osteoarthritis; Property: dim AND Title or Keywords: prevalence; Property: dim<br>(2) Title or Keywords: osteoarthritis; Property: dim AND Title or Keywords: incidence; Property: dim<br>(3) Title or Keywords: osteoarthritis; Property: dim AND Title or Keywords: epidemiology; Property: dim

Supplementary Table 2. Quality Scores of Individual Studies

	Evaluation Standard						
First author	(1)	(2)	(3)	(4)	(5)	(6)	total
Zhang et al	1	1	0	1	1	0	4
Huang et al	1	1	1	0	1	0	4
Tang et al	1	1	1	0	1	1	5
Liu et al	1	1	1	1	1	1	6
Wang et al	1	1	0	1	1	1	5
Jiang et al	1	1	1	1	1	1	6
Kang et al	1	1	0	1	1	1	5
Du et al	1	1	0	1	1	1	5
Zhang et al	1	1	0	1	1	0	4
Tian et al	1	1	1	1	1	1	6
Xue et al	1	1	1	1	0	1	5
Li et al	1	1	1	1	0	0	4
Xiang et al	1	1	1	1	1	1	6
Xiang et al	1	1	1	1	1	1	6
Gu et al	1	0	1	1	0	1	4
Fan et al	1	1	1	1	0	0	4
Wang et al	1	1	1	1	1	1	6
Tang et al	1	1	0	1	1	0	4
Wang et al	1	1	0	1	1	1	5
Zang et al	1	1	0	1	1	1	5
Zeng et al	1	1	0	1	0	0	3

1. Was study design clearly described? 2. Does the study define the information source of the survey? 3. Does the study report the time period of patient inclusion? 4. Does the study report the diagnostic criteria of knee OA clearly? 5. Does participant selection procedures reported clearly? 6. Does the study summarize patient response rates and completeness of data collection?

Supplementary Table 3. Diagnostic Criteria of Symptomatic OA for Each Study

Name	Diagnostic Criteria of Symptomatic OA
Junfeng Zhang	American College of Rheumatology (ACR);KL grades; clinical symptoms;X-ray examination
Keqiang Huang	Repeated knee pain within the past month, presence of at least one of three factors (age >50 years, morning stiffness less than 30 minutes, or crepitus in knee movement), and X-ray showing signs of joint clearance narrowing, subchondral sclerosis, subchondral cysts, or osteophyte formation.
Xu Tang	Participants were first asked whether they were often "troubled" with any body pain. Each participant was asked whether he or she had physiciandiagnosed arthritis. We defined a subject as having symptomatic knee OA if he or she responded positively to both questions. The questions used to assess symptomatic knee OA are shown in an appendix available on the Arthritis & Rheumatology web site at http://onlinelibrary.wiley.com/doi/10.1002/art.39465/abstract.
Yuan Liu	Symptomatic knee OA was defined as having at least one knee with both a K/L grade 2 and a positive response to the question, "In the past 12 months, have you had knee pain lasting most days for at least a month?"
Yuewen Wang	Kellgren & Lawrence OA grading;Clinical criteria for the fnal diagnosis of primary OA
Liying Jiang	A subject was characterized as having radiographic knee OA if either knee met the KellgrenLawrence Scoring System.Symptomatic knee OA required the presence of these radiographic findings plus 1 month of knee pain during the previous 12 months.
Xiaozheng Kang	A subject was characterized as having radiographic knee OA if either knee met the KellgrenLawrence Scoring System.Symptomatic knee OA required the presence of these radiographic findings plus 1 month of knee pain during the previous 12 months.
Hui Du	(1)Questionnaire. The first question was ''Have you ever had pain in or around a knee on most days for at least a month?'' as mentioned above. (2)Radiography. K/L grading system
Yanfeng Zhang	(1)knee pain; (2)Joint movements have bone sounds; (3)morning stiffness; (4)age≥38 years old; (5)bony hypertrophy. It satisfies (1),(2),(3),(4) conditions or (1),(2),(5) conditions or (1),(4),(5) conditions.
Shaoqi Tian	(1)knee pain; (2)Joint movements have bone sounds; (3)morning stiffness; (4)age≥38 years old; (5)bony hypertrophy. It satisfies (1),(2),(3),(4) conditions or (1),(2),(5) conditions or (1),(4),(5) conditions.
Qingyun Xue	Repeated knee pain within the past month, presence of at least one of three factors (age >50 years, morning stiffness less than 30 minutes, or crepitus in knee movement), and X-ray showing signs of joint clearance narrowing, subchondral sclerosis, subchondral cysts, or osteophyte formation.
Yufei Li	KL grades; clinical symptoms;X-ray examination
Xiaowei Xiang	(1)knee pain; (2)Joint movements have bone sounds; (3)morning stiffness; (4)age≥38 years old; (5)bony hypertrophy. It satisfies (1),(2),(3),(4) conditions or (1),(2),(5) conditions or (1),(4),(5) conditions.
Zhenyong Xiang	KL grades; clinical symptoms;X-ray examination
Bin Gu	Clinical symptoms;X-ray examination
Yan Fan	KL grades; clinical symptoms;X-ray examination
Lei Wang	Repeated knee pain within the past month, presence of at least one of three factors (age >50 years, morning stiffness less than 30 minutes, or crepitus in knee movement), and X-ray showing signs of joint clearance narrowing, subchondral sclerosis, subchondral cysts, or osteophyte formation.
Minsheng Tang	KL grades; clinical symptoms;X-ray examination
Wei Wang	KL grades; clinical symptoms;X-ray examination
Changhai Zang	KL grades; clinical symptoms;X-ray examination
Qingyu Zeng	Clinical symptoms;X-ray examination

Supplementary Table 4. Analysis of The Publication Bias

Groups	Subgroups	t value	p value	95%CI
Overall Pooled Studies		0.030	0.976	-0.215~0.222
Study Year	1990≤year≤2008:	0.729	0.506	-28.936~49.539
	2008＜year≤2013	0.929	0.389	-25.302~11.380
	year＞2013	1.285	0.255	-12.902~38.707
Subgroup of Age	40-49 years old	1.239	0.247	-21.456~6.273
	50-59 years old	1.326	0.214	-36.290~9.205
	60-69 years old	0.844	0.417	-34.720~15.472
	over 70 years old	0.312	0.760	-36.585~27.419
Subgroup of Gender	Males	0.906	0.378	-24.872~9.973
	Females	0.894	0.385	-12.068~29.653
Subgroup of Area	Northern China	3.255	0.012	-21.676~-3.700
	Southern China	0.395	0.705	-43.818~61.392
Subgroup of Region	Rural	0.395	0.702	-17.077~11.997
	Urban	0.938	0.370	-12.564~30.824
	BMI≤24	0.869	0.449	-46.180~26.362
Subgroup of BMI	24＜BMI＜28	0.904	0.417	-43.055~21.909
	BMI≥28	0.811	0.463	-52.467~28.752
