# Supplementary material for: The Prevalence of Symptomatic Knee Osteoarthritis in Relation to Age, Sex, Area, Region, and Body Mass Index in China: A Systematic Review and Meta-Analysis
Source: Front Med (Lausanne). 2020 Jul 16;7:304. doi: 10.3389/fmed.2020.00304 (PMC7378378; doi:10.3389/fmed.2020.00304)
Supplement: Supplementary file 2 [file Table_1.DOC]

| **Section/topic** | **#** | **Checklist item** | **Reported on page #** |
| --- | --- | --- | --- |
| **TITLE** | | |  |
| Title | 1 | The prevalence of symptomatic knee osteoarthritis in relation to age, sex, area, region, and body mass index in China: a systematic review and meta-analysis | 1 |
| **ABSTRACT** | | |  |
| Structured summary | 2 | *Objective:* This study aimed to investigate the overall prevalence of symptomatic knee osteoarthritis (OA) in China by conducting a meta-analysis.  *Methods:* Six databases were searched to identify studies published before October 1, 2017. The search terms included “osteoarthritis,” “prevalence” or “incidence” or “epidemiology,” AND “China” or “Chinese.” The χ^2^-based Q statistic and *I*^2^ metrics were used for exploring the sources of heterogeneity. Random models were utilized to obtain prevalence estimates due to the heterogeneity that was observed. Comprehensive Meta-Analysis version 2.0 was used for assessing publication bias by inspecting funnel plots and Egger’s tests.  *Results:* Twenty-one eligible studies (74,908 participants in total) were identified. The overall pooled prevalence of symptomatic knee OA in China was 14.6%. The prevalence of symptomatic knee OA presented a rapid growth trend between the periods of 1990–2008 and 2008–2013 (9.1% vs. 20.1%, *p* = 0.005). However, after 2013, the prevalence dropped to 14.9% (*p* = 0.01). The prevalence rates of symptomatic knee OA increased with age and presented an almost linear growth after 40 years of age. Compared with males (10.9%), females (19.1%) exhibited a higher prevalence of symptomatic knee OA (*p* = 0.015). The symptomatic knee OA prevalence was significantly higher in rural than it was in urban areas (16.9% vs. 11.1%, *p* = 0.037).  *Conclusion:* For symptomatic knee OA intervention, more attention should be paid to females, people in rural areas, and people aged over 40 years. | 3 |
| INTRODUCTION | | |  |
| Rationale | 3 | China is comprised of 34 province-level administrative regions with a population of 1.4 billion people. A systematic reviews on the prevalence of symptomatic knee OA in Chinese population was lacking. | 4,5 |
| Objectives | 4 | To investigate the prevalence of symptomatic knee osteoarthritis (OA) in China by conducting a meta-analysis. | 3 |
| **METHODS** | | |  |
| Protocol and registration | 5 |  |  |
| Eligibility criteria | 6 | The criteria for inclusion were as follows:  (1) The study was a population-based survey with a representative sample of Chinese or mixed populations;  (2) The investigation involved random or cluster sampling;  (3) The sample size was >500;  (4) The diagnostic criteria were clear;  (5) The prevalence/incidence of symptomatic knee OA was evaluated and displayed clearly or could be calculated;  (6) Important information, including authors, age, sex, region, city, and year, was clear; and  (7) Studies conducted in the general population rather than among special groups ([pregnant](../../../../F:%5CProgram%20Files%5CYoudao%5CDict%5C7.5.2.0%5Cresultui%5Cdict%5C%3Fkeyword=pregnant) [women](../../../../F:%5CProgram%20Files%5CYoudao%5CDict%5C7.5.2.0%5Cresultui%5Cdict%5C%3Fkeyword=woman), diabetics, cardiovascular disease patients) were included. | 5,6 |
| Information sources | 7 | PubMed, EMBASE, the Web of Science, the China National Knowledge Infrastructure, the VIP Database for Chinese Technical Periodicals, and the Wan Fang Database for Chinese Periodicals | 5 |
| Search | 8 | Search terms included ‘osteoarthritis’, ‘prevalence’ or ‘incidence’ or ‘epidemiology’, AND ‘China’ or ‘Chinese’. | 5 |
| Study selection | 9 | Note Express was applied to remove duplicates. The title and abstract of all studies were screened independently by two reviewers against the inclusion and exclusion criteria. Articles that did not meet the inclusion criteria were excluded. Full-text articles were assessed for eligibility. | 5,6 |
| Data collection process | 10 | The articles included in this study were reviewed independently by two investigators, who also extracted and evaluated the quality of the data. | 6 |
| Data items | 11 | (1) first author; (2) study year; (3) province; (4) region (urban or rural); (5) overall sample size; (6) response rate; (7) the method used to sample subjects; (8) diagnostic criteria of symptomatic knee OA; and (9) the prevalence rates obtained for the overall sample and subgroups | 6 |
| Risk of bias in individual studies | 12 | Egger's test and Funnel plots were made by Comprehensive Meta-Analysis version 2.0. No publication bias exists if the studies are arranged symmetrically around the central line with a p value of >0.05. | 7,8 |
| Summary measures | 13 | The prevalence and their associated 95% confidence intervals (CIs) | 7 |
| Synthesis of results | 14 | Comprehensive Meta-Analysis version 2.0 was applied to estimate the prevalence rates of symptomatic knee OA, with 95% confidence intervals (CIs). | 7 |

Page 1 of 2

| **Section/topic** | **#** | **Checklist item** | **Reported on page #** |
| --- | --- | --- | --- |
| Risk of bias across studies | 15 | To examine the authenticity of data, Egger's test and Funnel plots were made by Comprehensive Meta-Analysis version 2.0. No publication bias exists if the studies are arranged symmetrically around the central line with a p value of >0.05. | 7 |
| Additional analyses | 16 | The statistical significant differences between the subgroups were evaluted by Kruskal-Wallis test and Mann-Whitney test. The Z value and p vallue were obtained and a value of p<0.05 was considered statistically significant. | 7,8 |
| RESULTS | | |  |
| Study selection | 17 | Supplementary Figure 1 | Supplementary File |
| Study characteristics | 18 | Table1 | Table1 |
| Risk of bias within studies | 19 | N/A | N/A |
| Results of individual studies | 20 | Figure1-5 | Figure1-5 |
| Synthesis of results | 21 | Figure1-5 | Figure1-5 |
| Risk of bias across studies | 22 | Supplementary Figure 7-23 | Supplementary Figure 7-23 |
| Additional analysis | 23 | Results | 6-9 |
| **DISCUSSION** | | |  |
| Summary of evidence | 24 | This systematic review and meta-analysis first estimated the prevalence of symptomatic knee osteoarthritis in China. In the meta-analyses, 21 studies (74,908 people) on symptomatic knee osteoarthritis were included. | 8-11 |
| Limitations | 25 | First, the included studies did not cover all the cities in China equally. Second, due to the heterogeneity among the papers, our sample changed from variable to variable. | 15 |
| Conclusions | 26 | The overall pooled prevalence of symptomatic knee OA was 14.6% (95% CI = 11.4–18.5%) in China. The prevalence rates of symptomatic knee OA presented an almost linear growth after 40 years old. Females exhibited a higher prevalence of symptomatic knee OA than males did, while the prevalence was significantly higher in rural than it was in urban areas. There was no statistically significant difference between northern China and southern China. The prevalence of symptomatic knee OA did not rise significantly as the BMI increased. More attention should be paid to females, people in rural areas, and people aged over 40 for the symptomatic knee OA intervention. More nationally focused and accurate investigations of the prevalence of symptomatic knee OA are needed. | 16 |
| **FUNDING** | | |  |
| Funding | 27 | This work was supported by Shanghai Sailing Program (18YF1403500) and Shanghai Municipal Commission of Health and Family Planning (20174Y0169). | 16 |

*From:*  Moher D, Liberati A, Tetzlaff J, Altman DG, The PRISMA Group (2009). Preferred Reporting Items for Systematic Reviews and Meta-Analyses: The PRISMA Statement. PLoS Med 6(6): e1000097. doi:10.1371/journal.pmed1000097

For more information, visit: **www.prisma-statement.org**.

Page 2 of 2
